# Supplementary material for: Integrating High-Value Cost-Conscious Care into an Existing Medical School Curriculum
Source: MedEdPORTAL. 2025 Jan 28;21:11490. doi: 10.15766/mep_2374-8265.11490 (PMC11772531; doi:10.15766/mep_2374-8265.11490)
Supplement: Supplementary file 1 — Clinical Informatics Pearl 1.docxClinical Informatics Pearl 2.docxClinical Informatics Pearl 3.docxGamified Clinical Skills Lab.pptxCost Worksheet.docxFacilitator Guide.docxPre- and Postsurvey.docx [file mep_2374-8265.11490-s001.zip › A. Clinical Informatics Pearl 1.docx]

**Clinical Informatics Pearls 1**

(Approximate time for activity: 15 minutes)

Curriculum integration instruction: This is the first assignment in our three-part Clinical Informatics Pearl series, offering essential instructions and a clinical scenario for medical students to complete it. It is best implemented during or after their lessons on imaging modalities and dementia, and before starting their clinical rotations. An instructional video can be created using the provided sample transcript, though a written version is also a viable alternative.

This week’s assignment focuses on how to use readily available imaging appropriateness resources such as the American College of Radiology Appropriateness Criteria [(ACR)](https://www.acr.org/Clinical-Resources/ACR-Appropriateness-Criteria)^1^ or [U.S. Preventive Services Task Force recommendations](https://www.uspreventiveservicestaskforce.org/uspstf/recommendation-topics) (can use any resource) to make decisions about the best value imaging to order.

This is an example of a transcript that can be used when making the Pearls (can use any resource):

Hi I'm [Presenter]. In this Pearl, we will be going over how to utilize the American College of Radiology appropriateness criteria for imaging recommendations. Okay, so let's say we're debating whether or not imaging is indicated for a patient that's coming in with a new headache. The first step to utilize this resource is just to google American College of Radiology appropriateness criteria. Then, click on the first link, scroll down to explore by scenario, and narrow down the search to just headaches by typing "headache." We know the patient is coming in with a new headache, so we click on this clinical scenario. Now we have a list of whether or not imaging is appropriate. We can look at the adult recommendations, the pediatric recommendations, and all the different types of imaging and whether or not they're recommended through this criteria. This is a great resource to utilize when figuring out whether or not imaging is indicated. Thank you.

**Objectives:**

- Successfully navigate the [Insert imaging appropriateness resource] site to determine if a specific diagnostic test is appropriate for a particular diagnosis.

**Instructions:** [See example instructions below using ACR as a resource]

1. After viewing this week’s Pearl, please have the [ACR site](https://www.acr.org/Clinical-Resources/ACR-Appropriateness-Criteria) open in a browser.
2. Review John’s chart (below) fully.
   1. Search the ACR site to determine which CT scan would be appropriate to determine a possible diagnosis of dementia for John. **Hint – Try using dementia for your search.** Take a screen shot of your findings and submit them to the online assignment portal.

**HPI**: John is a 72 y.o. man presenting to an outpatient clinic complaining of progressive forgetfulness and sleep disturbances that have been occurring over the past 6 months. He initially noticed difficulty remembering where he placed items and appointments, which has gradually worsened. His family describes that he often repeats stories. The patient endorses difficulty falling asleep. He denies any headache or vision changes. He denies any recent trauma.

**PMH**: Type II Diabetes Mellitus, HTN, Anxiety

**Medications**: Metformin, Lisinopril

**Social History:** Non-smoker, occasional EtOH. Retired. Lives with wife at home.

**Vitals**: Temperature 37.3 C, HR 78 bpm, BP 135/82, SPO2 100%

**Physical Exam:**

General: Elderly gentleman in no acute distress.

Head: Atraumatic. Normocephalic

Eyes: PERRL. EOMI. No icterus.

Heart: Regular rate and rhythm. No murmurs.

Lungs: Clear to auscultation bilaterally

MSK: Moves all 4 extremities. No edema.

Neuro: Alert and oriented x. CN 2-12 are intact, Strength 5/5 bilateral upper and lower extremities, Reflexes are normal at patella/biceps/achilles bilaterally, sensation intact to light touch throughout

**Recent Labs:** HA1C: 6.8%, CBC: Unremarkable, CMP: Unremarkable

**Anticipated Answer/Discussion:**

Anticipated Answer: Based on the clinical scenario and using resources such as ACR, the anticipated answer would be either “MRI head without IV contrast” or “CT head without IV contrast” to evaluate for structural causes of dementia.

Discussion Point: While MRI provides detailed imaging, it takes longer to complete compared to a CT scan. This duration is a crucial factor in decision-making, especially considering clinical circumstances. For instance, if we anticipate that John might struggle to remain still for the 15-90 minutes required for an MRI, opting for a CT scan could be more practical.

**References**

1. American College of Radiology. ACR Appropriateness Criteria. American College of Radiology. Accessed 2024. https://www.acr.org/Clinical-Resources/ACR-Appropriateness-Criteria
2. U.S. Preventive Services Task Force. Recommendation Topics. U.S. Preventive Services Task Force. Accessed 2024. https://www.uspreventiveservicestaskforce.org/uspstf/recommendation-topics
