# Supplementary material for: Integrating High-Value Cost-Conscious Care into an Existing Medical School Curriculum
Source: MedEdPORTAL. 2025 Jan 28;21:11490. doi: 10.15766/mep_2374-8265.11490 (PMC11772531; doi:10.15766/mep_2374-8265.11490)
Supplement: Supplementary file 1 — Clinical Informatics Pearl 1.docxClinical Informatics Pearl 2.docxClinical Informatics Pearl 3.docxGamified Clinical Skills Lab.pptxCost Worksheet.docxFacilitator Guide.docxPre- and Postsurvey.docx [file mep_2374-8265.11490-s001.zip › B. Clinical Informatics Pearl 2.docx]

**Clinical Informatics Pearls 2**

(Approximate time for activity: 15 minutes)

Curriculum integration instruction: This is the second assignment in our three-part Clinical Informatics Pearl series, providing essential instructions and a clinical scenario for medical students to complete it. It can be delivered 1-5 weeks after the first assignment. Given that this activity relates to schizophrenia, it is best integrated once students are introduced to psychiatric disorders. An instructional video can be created using the provided sample transcript, but a written version is also an acceptable alternative.

The assignment this week utilizes readily available medication cost resources such as [Good RX^1^](https://www.goodrx.com/) or [RxSaver](https://www.rxsaver.com/)^2^ (can use any resource) and the University Hospital’s Pricing list to help understand cost. If the institution does not have a pricing list, the [Fair Health Consumer Cost Look-Up](https://www.independenthealth.com/individuals-and-families/tools-forms-and-more/health-tools/fair-health-cost-lookup)^3^ can also be used. The goal of this activity is to learn how to check on the costs of medications or diagnostic testing before ordering.

This is an example of a transcript that can be used when making the Pearls (can use any resource):

Hi, I'm [Presenter]. In this Pearl, I'll be going over two resources that you can use to check the prices of medications, procedures, or labs. The first resource is called GoodRx. You can find it by simply typing "GoodRx" into Google, and it will be the first result. Next, you'll type in the medication you're interested in, and for this example, we can look at Lamictal. The first step is making sure your prescription matches the correct form, dosage, and quantity. Once that's entered, you can update it to see the different prices at nearby pharmacies, allowing you to compare prices and discuss them with your patients. Another feature is checking if a medication is covered by Medicare by clicking "More" and selecting "Medicare" for further details. You can also explore drug info, scroll down to "Alternatives," and compare similar drugs, considering their pros, cons, and prices—another helpful option for patient discussions. The next resource we can use is to check lab and procedure costs within the University Hospital Pricing List. [Description unique to each hospital system].

**Objectives:**

- Effectively perform chart review
- How to find costs of diagnostic tests and medications in regard to value-based care

**Instructions:** [See example instructions below using GoodRx as a resource]

1. Review this week’s Pearl video.
2. Thoroughly review your patient’s note below
3. Head imaging might be warranted, but you want to see comparative costs first to provide counseling given patients concerns about cost.
   1. [Insert school specific instructions on how to access University Lab/Imaging Cost Spreadsheet]
   2. Find cost of Brain MRI with contrast and a CT head with contrast on University Hospital’s Pricing Sheet
   3. Would you recommend head imaging in this situation? State your choice and why. Consider checking the [ACR](https://www.acr.org/Clinical-Resources/ACR-Appropriateness-Criteria)^4^ appropriateness criteria to help bolster the reasoning for your choice.
4. Medications are also considered for her hallucinations.
   1. Navigate to GoodRx
      1. You are considering olanzapine, 5 mg tablet
      2. The patient indicates she normally uses Fred Meyer pharmacy, but could possibly switch to Walmart.
      3. Include screen shots if you can of your findings.
      4. State your choice of pharmacy and why.

**HPI:** Kay is a 29 y.o. female brought to the clinic by her husband for concerns about her behavior. For the past 8 months, patient has been expressing paranoid thoughts and concerns that someone is poisoning her food. She describes hearing radio signals that warn her about who is doing this. Her husband has also seen her describing objects in the room that are not there. Patient is currently uninsured and describes concerns about cost.

**PMH**: Anxiety

**Medications**: Fluoxetine

**Social History:** Non-smoker, no EtOH. Lives with her husband at home.

**Vitals**: Temperature 37 C, HR 70 bpm, BP 126/85, SPO2 100%

**Physical Exam:**

General: Anxious appearing female in no acute distress.

Head: Atraumatic. Normocephalic

Eyes: PERRL. EOMI. No icterus.

Heart: Regular rate and rhythm. No murmurs.

Lungs: Clear to auscultation bilaterally

MSK: Moves all 4 extremities. No edema.

Neuro: Alert and oriented x. CN 2-12 are intact, Strength 5/5 bilateral upper and lower extremities, Reflexes are normal at patella/biceps/achilles bilaterally, sensation intact to light touch throughout

Psych: Mood: Anxious. Affect: Flat. Appears disheveled. Thought process disorganized. Endorses paranoid delusions and responds to internal stimuli. Poor insight.

**Labs**: TSH within normal limits. B12 within normal limits.

**Anticipated Answer/Discussion:**

Anticipated Answer:

1. The cost of imaging will vary depending on the institution. This clinical scenario suggests a potential diagnosis of schizophrenia. Although schizophrenia is primarily diagnosed clinically, given Kay’s sudden onset of psychiatric symptoms at the age of 29, it would be important to conduct head imaging to rule out potential causes such as tumors, strokes, or infections. According to the American College of Radiology Appropriateness Criteria^4^, both CT and MRI scans are suitable options. However, CT scans are typically more cost-effective and quicker.
2. At this time, GoodRx offers a special coupon for Olanzapine at RiteAid, making it the most affordable option. While Walmart is slightly cheaper than Fred Meyer, I would recommend RiteAid. However, I will present all options to Kay for her consideration.

Discussion Point: Given Kay’s concerns about costs, it’s crucial to explore all available options for addressing her ongoing psychiatric symptoms, including the possibility of foregoing imaging. Hospitals and clinics often have social services that can assist in finding affordable imaging solutions or insurance coverage.

References

1. GoodRx. GoodRx website. Accessed 2024. https://www.goodrx.com/

2. RxSaver. Prescription Discounts and Coupons. RxSaver. Accessed 2024. https://www.rxsaver.com/

3. Fair Health Consumer. Healthcare Cost Estimator. FAIR Health Consumer. Accessed 2024. https://www.fairhealthconsumer.org/

4. American College of Radiology. ACR Appropriateness Criteria. American College of Radiology. Accessed 2024. https://www.acr.org/Clinical-Resources/ACR-Appropriateness-Criteria
