# Supplementary material for: Integrating High-Value Cost-Conscious Care into an Existing Medical School Curriculum
Source: MedEdPORTAL. 2025 Jan 28;21:11490. doi: 10.15766/mep_2374-8265.11490 (PMC11772531; doi:10.15766/mep_2374-8265.11490)
Supplement: Supplementary file 1 — Clinical Informatics Pearl 1.docxClinical Informatics Pearl 2.docxClinical Informatics Pearl 3.docxGamified Clinical Skills Lab.pptxCost Worksheet.docxFacilitator Guide.docxPre- and Postsurvey.docx [file mep_2374-8265.11490-s001.zip › C. Clinical Informatics Pearl 3.docx]

**Clinical Informatics Pearls 3**

(Approximate time for activity: 15 minutes)

Curriculum integration instruction: This is the third assignment in our three-part Clinical Informatics Pearl series, providing essential instructions and a clinical scenario for medical students to complete it. It can be delivered 1-5 weeks after the second assignment. Since this activity focuses on pediatric viral infections, it is best integrated after students have covered common viral infections in this population. An instructional video can be created using the provided sample transcript, but a written version is also an acceptable alternative.

You will use readily available cost-effective care guidelines such as [Choosing Wisely guidelines^1^](https://www.aafp.org/pubs/afp/collections/choosing-wisely.html) to help you determine when testing may or may not be indicated.

This is an example of a transcript that can be used when making the Pearls (can use any resource):

Hi, I'm [Presenter]. In this Pearl, I'll be going over resources that you can use to determine guidelines for certain medical tests. The resource we will be looking at today are the “Choosing Wisely guidelines.” You can find this by navigating to the American Academy of Family Physicians Choosing Wisely Recommendations. From here, you can select the discipline focus area you are searching for and browse through the guidelines.

**Objectives:**

- Use the cost-effective care guidelines to determine test necessity

**Instructions:** [See example instructions below using Choosing Wisely as a resource]

1. Review the patient’s chart below, as well as the pediatric Choosing Wisely Guidelines: https://www.aafp.org/pubs/afp/collections/choosing-wisely.html.
2. Determine whether or not you need to order a viral respiratory panel for this patient. Provide your answer and justification.

**HPI**: Lucas is a 5 y.o boy brought into the pediatric clinic for a cough in October. For the past three days, patient has had a productive cough. He is afebrile. The patients mother notes that the patient has been more fatigued but has been eating and drinking normally. Patient attends kindergarten and several other children in his class have similar symptoms.

**PMH**: None

**Medications**: None

**Vitals**: Temperature 37.0 C, Blood Pressure 100/60, HR 90, RR 20, SPO2 100% on room air

**Physical Exam:**

General: Pleasant 5 y.o. boy in no acute distress.

Head: Atraumatic. Normocephalic

Eyes: PERRL. EOMI. No icterus.

ENT: TMs normal bilaterally. Mild erythema in oropharynx. No tonsillar enlargement. Clear rhinorrhea from nares.

Heart: Regular rate and rhythm.

Lungs: Clear to auscultation bilaterally

MSK: Moves all 4 extremities. No edema.

Neuro: Alert. Non-focal.

**Anticipated Answer/Discussion:**

Anticipated Answer: Based on the "Choosing Wisely" guidelines^1^, it would generally not be recommended to order a broad viral respiratory panel for Lucas. The guidelines suggest avoiding routine broad respiratory pathogen panels unless the results directly impact patient management. Instead, they recommend using specific rapid tests for common pathogens, such as respiratory syncytial virus (RSV) or influenza, especially if these tests can expedite management decisions.

Discussion point: In Lucas’s case, given his mild symptoms, normal vital signs, and the fact that he is afebrile and in no acute distress, it might be more appropriate to monitor his symptoms and consider specific rapid tests if his condition changes or if there is a strong clinical suspicion of a particular pathogen.

**References**

1. American Academy of Family Physicians. Choosing Wisely Recommendations. AAFP website. Accessed 2024. https://www.aafp.org/pubs/afp/collections/choosing-wisely.html
