# Supplementary material for: Integrating High-Value Cost-Conscious Care into an Existing Medical School Curriculum
Source: MedEdPORTAL. 2025 Jan 28;21:11490. doi: 10.15766/mep_2374-8265.11490 (PMC11772531; doi:10.15766/mep_2374-8265.11490)
Supplement: Supplementary file 1 — Clinical Informatics Pearl 1.docxClinical Informatics Pearl 2.docxClinical Informatics Pearl 3.docxGamified Clinical Skills Lab.pptxCost Worksheet.docxFacilitator Guide.docxPre- and Postsurvey.docx [file mep_2374-8265.11490-s001.zip › D. Gamified Clinical Skills Lab.pptx]

## Slide 1
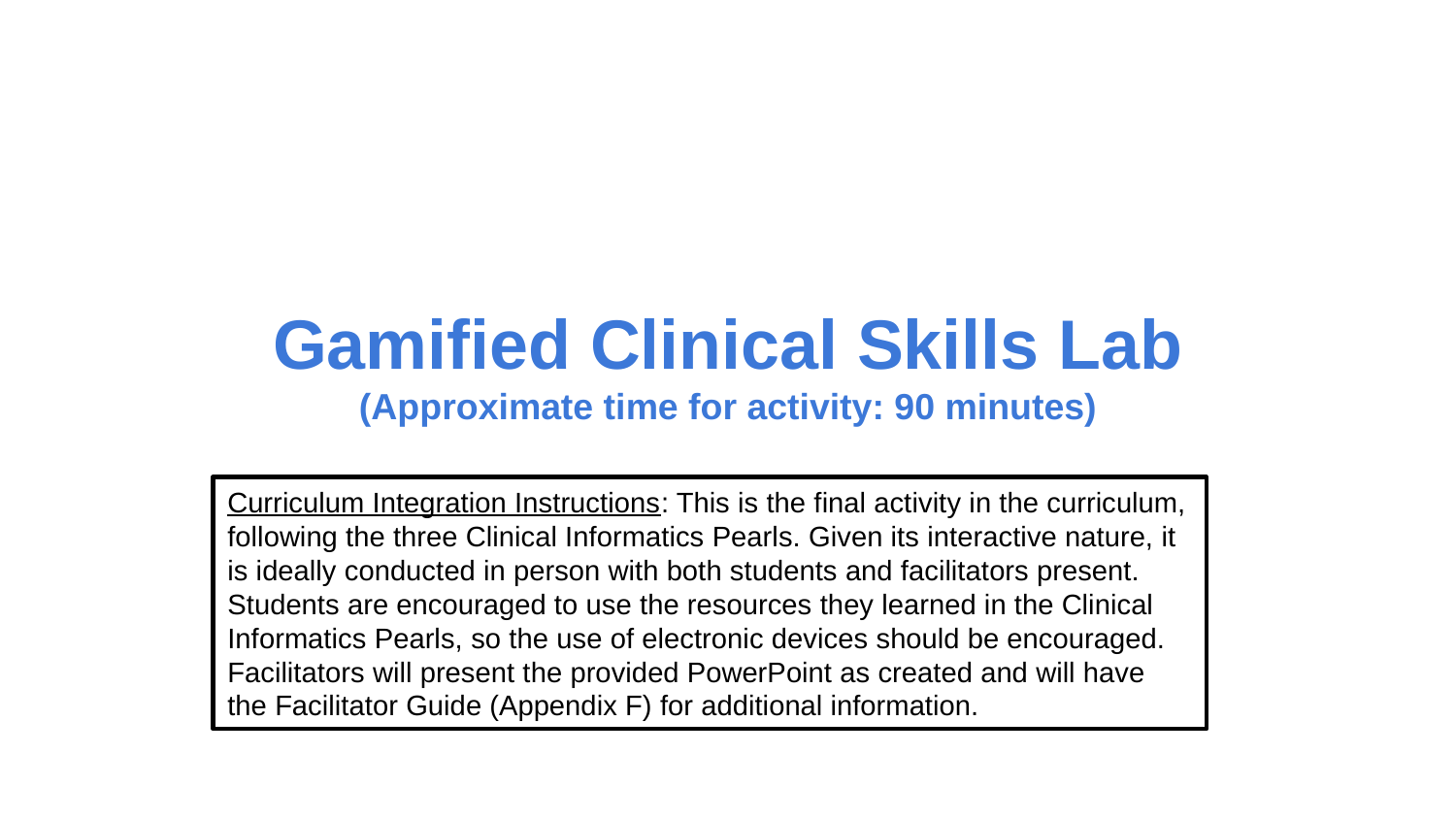

# Gamified Clinical Skills Lab (Approximate time for activity: 90 minutes)
Curriculum Integration Instructions: This is the final activity in the curriculum, following the three Clinical Informatics Pearls. Given its interactive nature, it is ideally conducted in person with both students and facilitators present. Students are encouraged to use the resources they learned in the Clinical Informatics Pearls, so the use of electronic devices should be encouraged. Facilitators will present the provided PowerPoint as created and will have the Facilitator Guide (Appendix F) for additional information.

## Slide 2
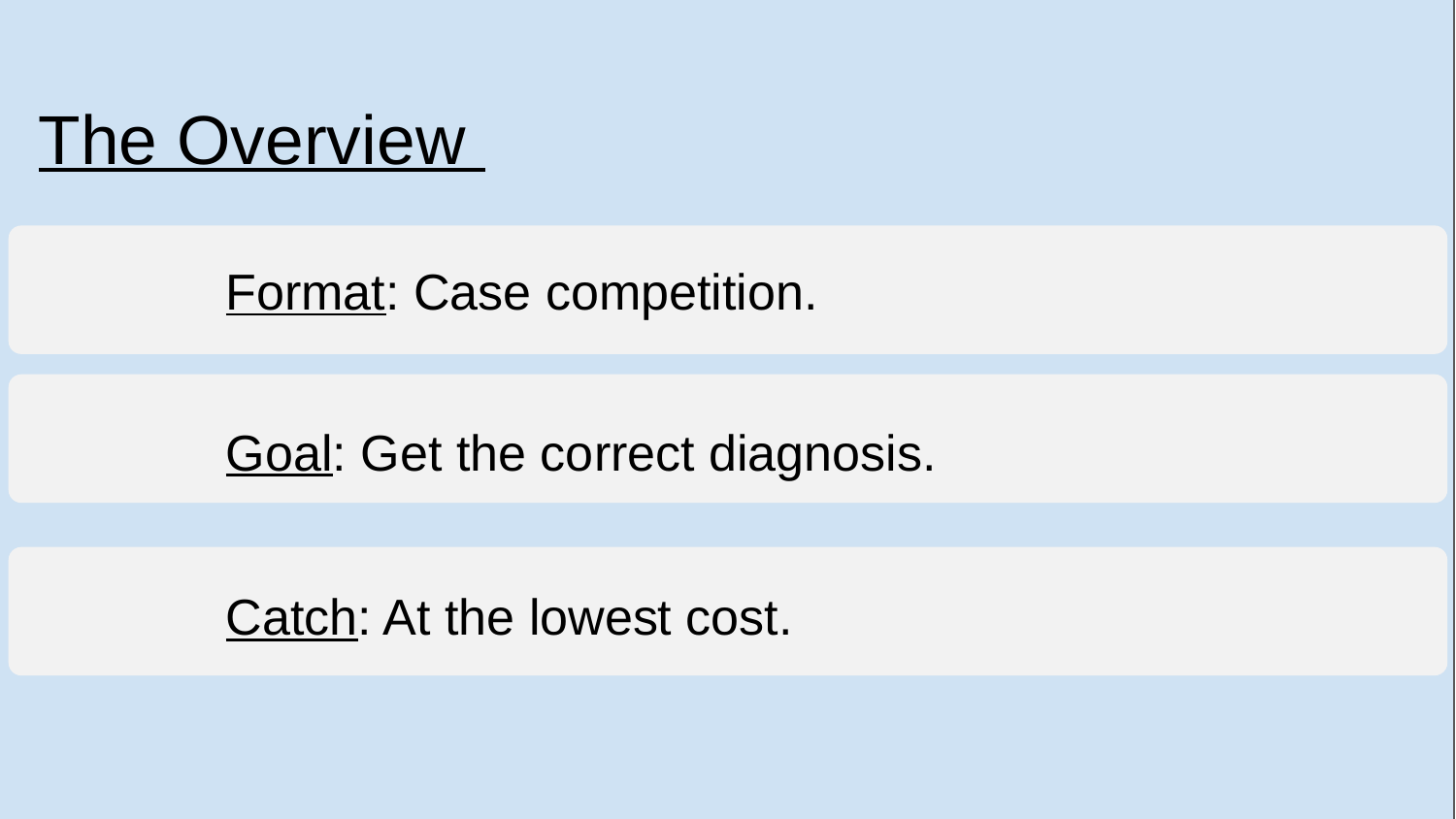

# The Overview
Format: Case competition.
Goal: Get the correct diagnosis.
Catch: At the lowest cost.

## Slide 3
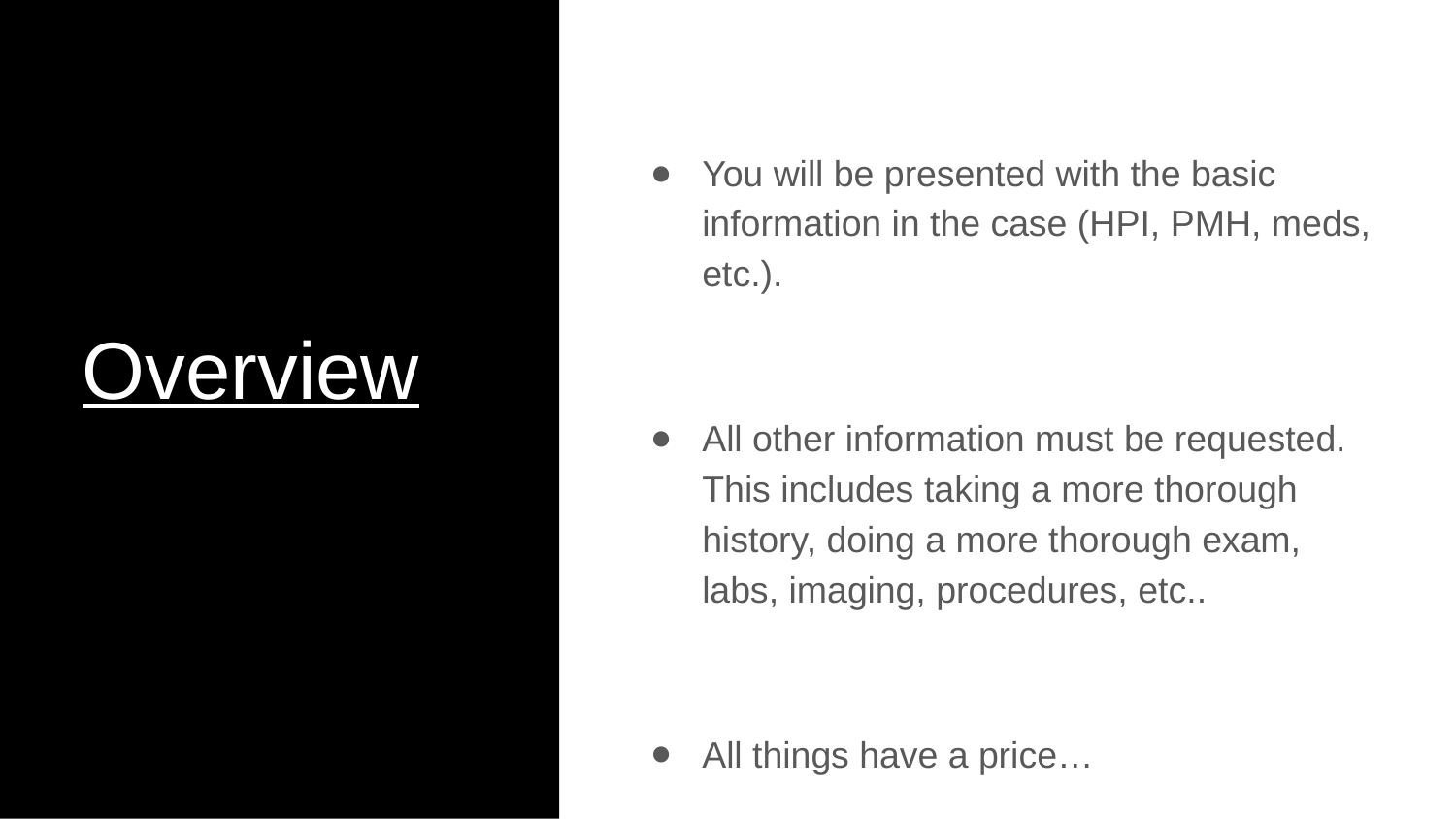

You will be presented with the basic information in the case (HPI, PMH, meds, etc.).
All other information must be requested. This includes taking a more thorough history, doing a more thorough exam, labs, imaging, procedures, etc..
All things have a price…
# Overview

## Slide 4
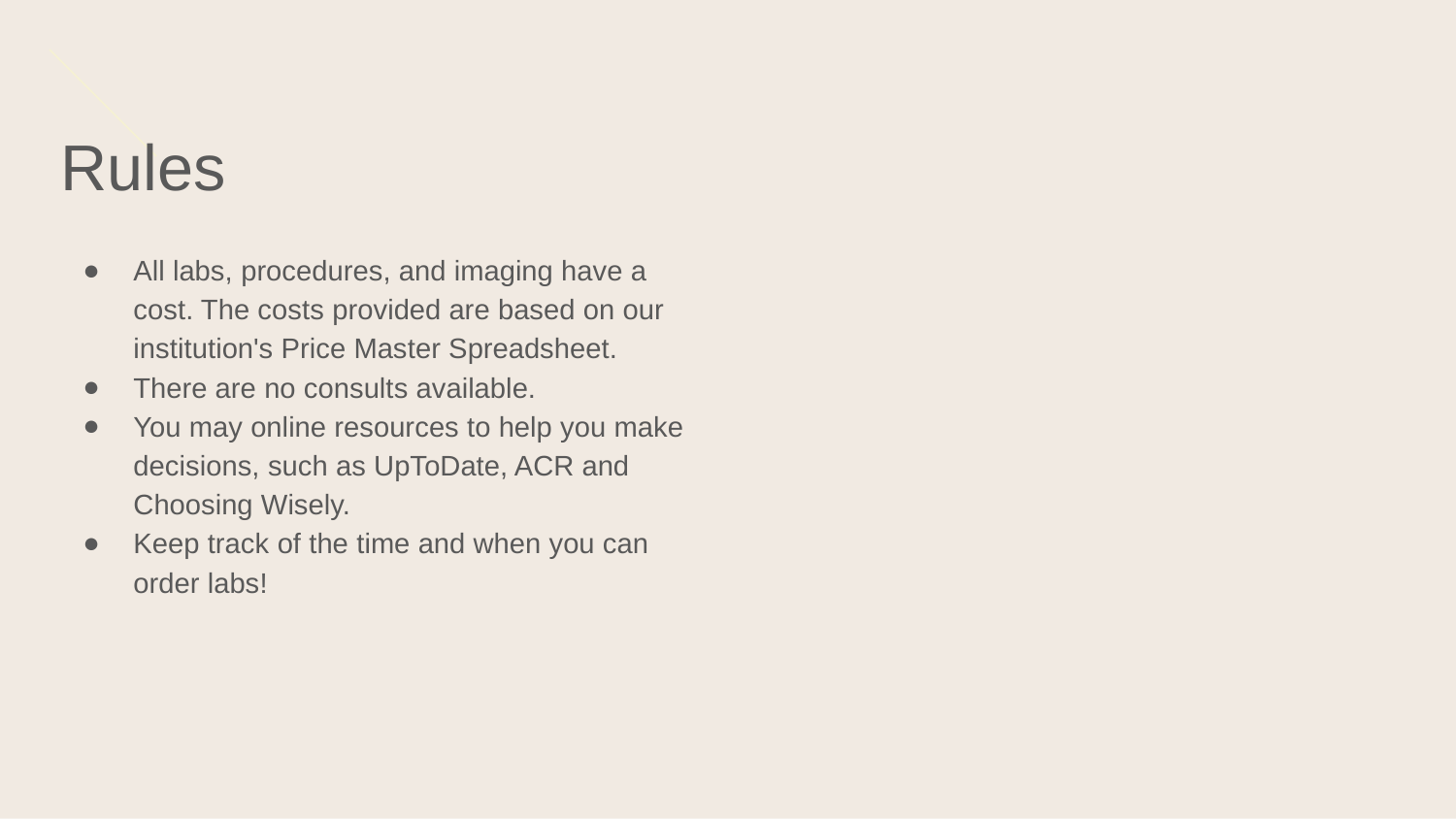

# Rules
All labs, procedures, and imaging have a cost. The costs provided are based on our institution's Price Master Spreadsheet.
There are no consults available.
You may online resources to help you make decisions, such as UpToDate, ACR and Choosing Wisely.
Keep track of the time and when you can order labs!

## Slide 5
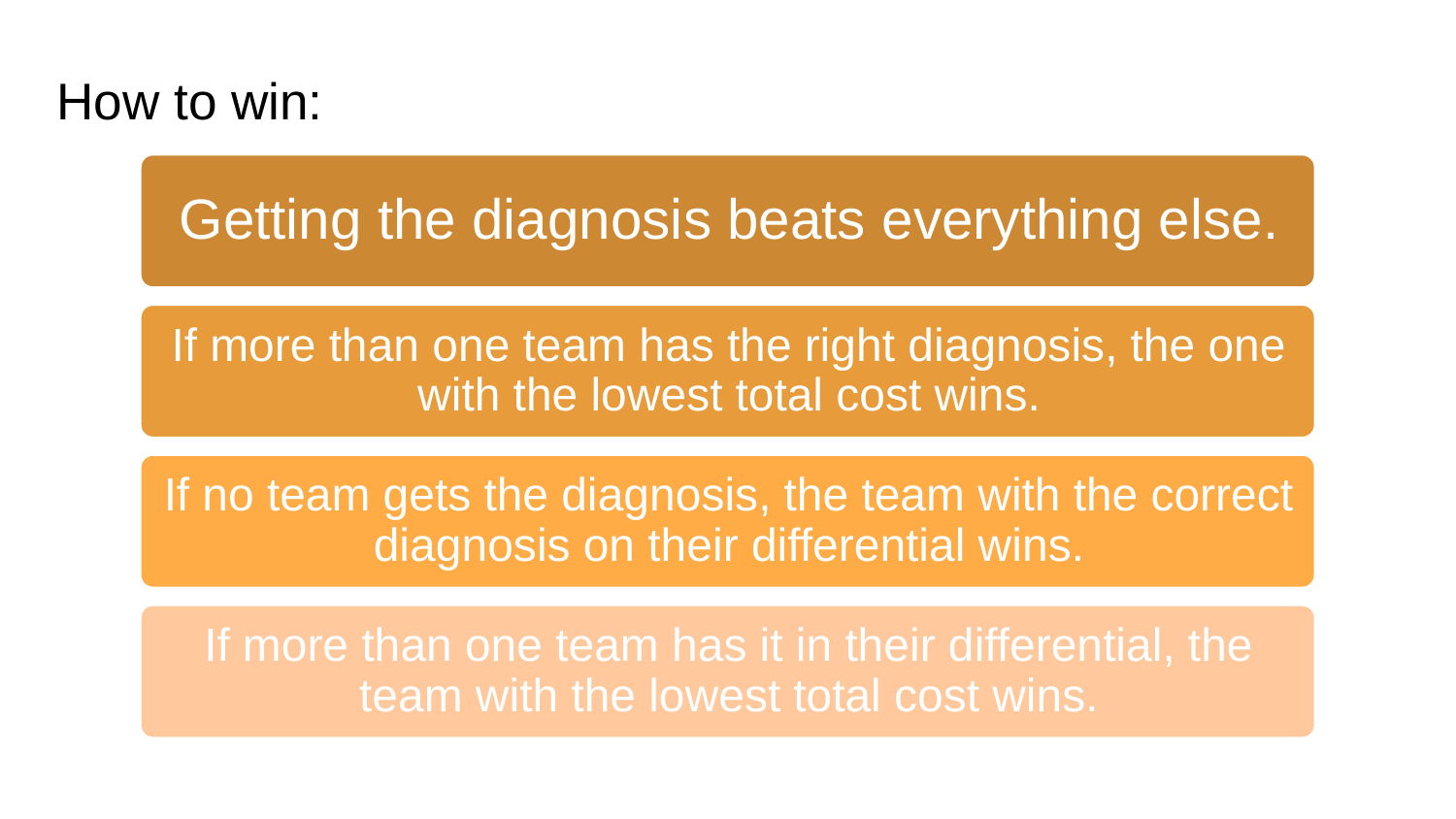

# How to win:

## Slide 6
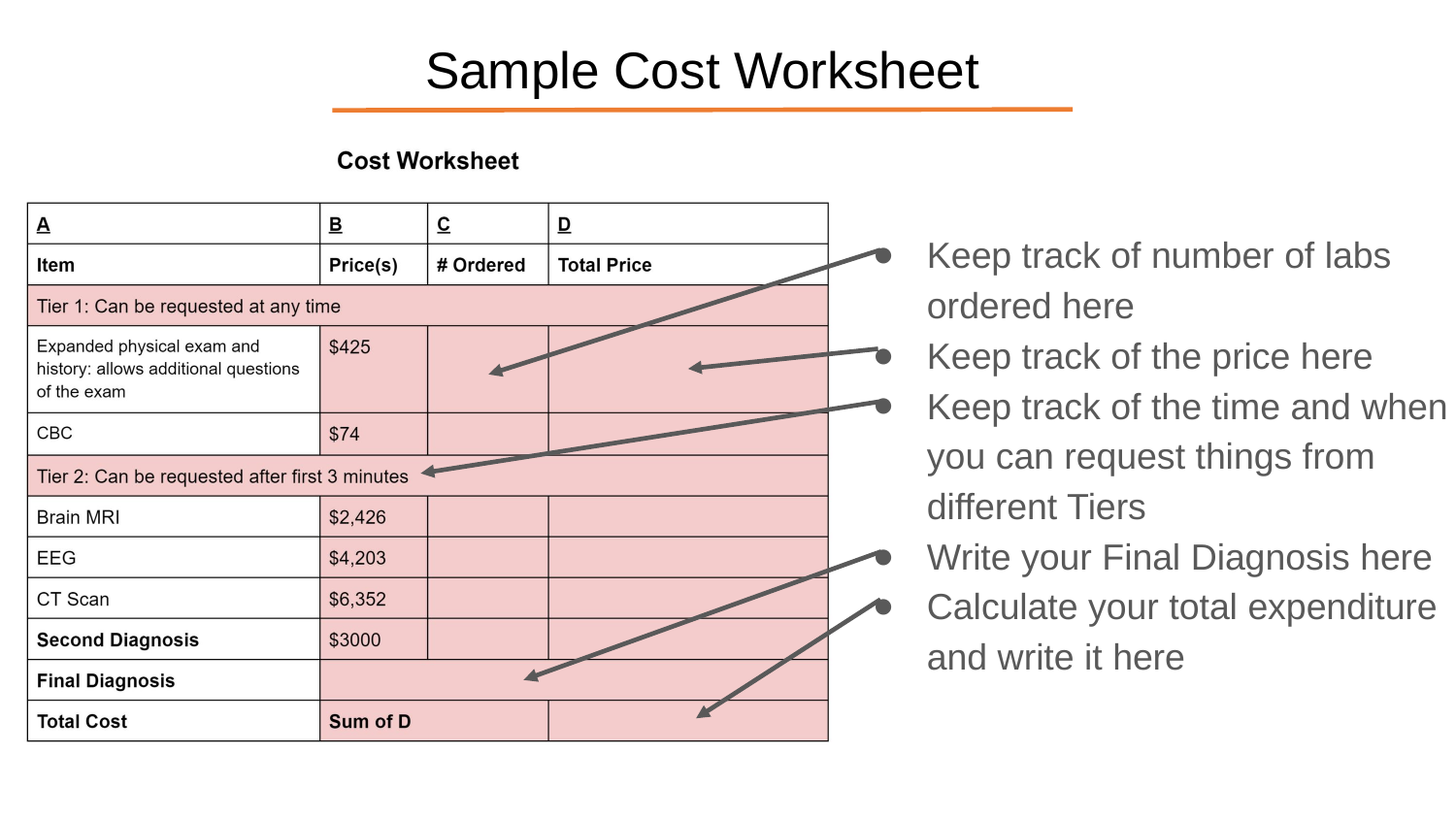

# Sample Cost Worksheet
Keep track of number of labs ordered here
Keep track of the price here
Keep track of the time and when you can request things from different Tiers
Write your Final Diagnosis here
Calculate your total expenditure and write it here

## Slide 7
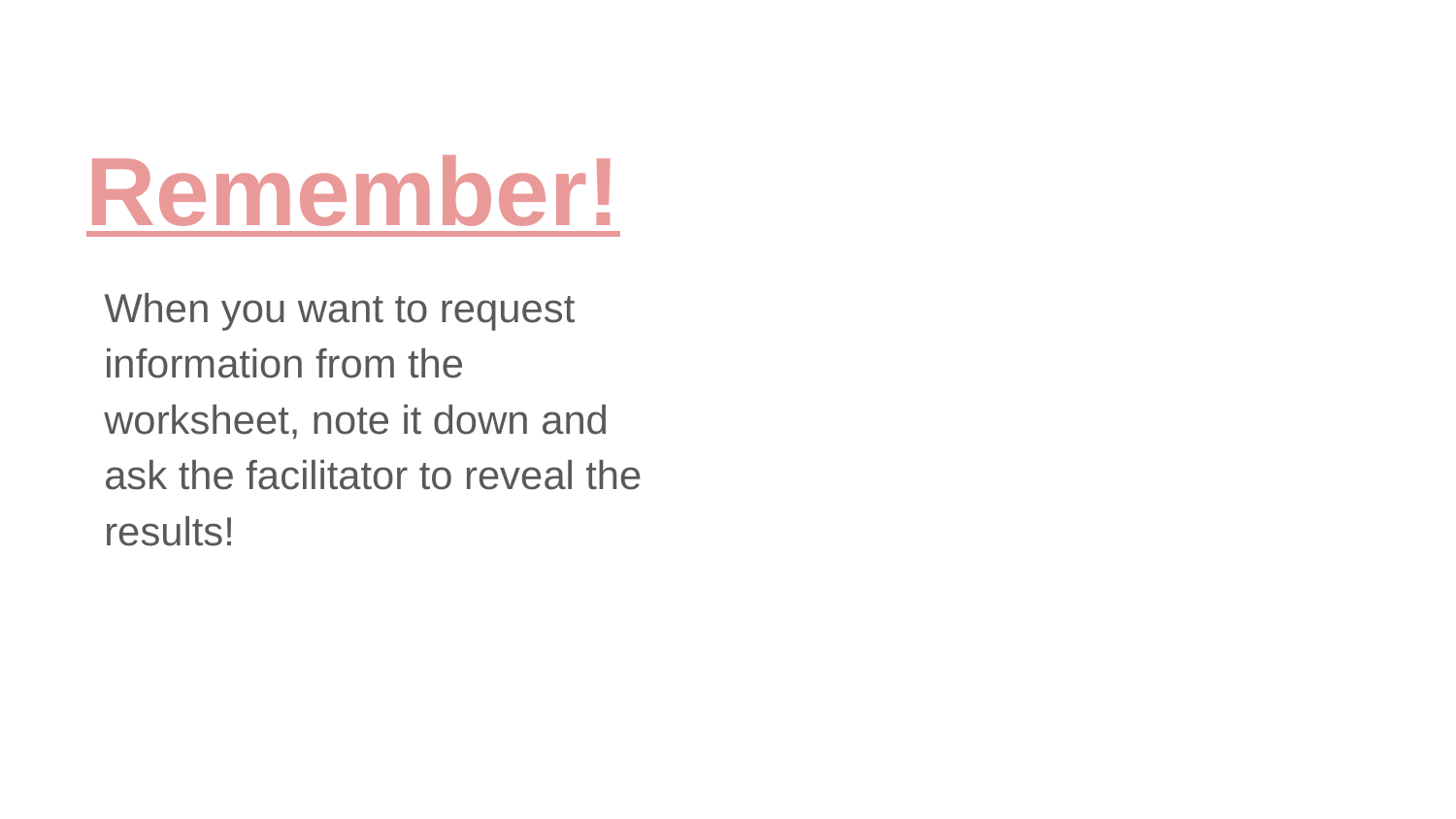

# Remember!
When you want to request information from the worksheet, note it down and ask the facilitator to reveal the results!

## Slide 8
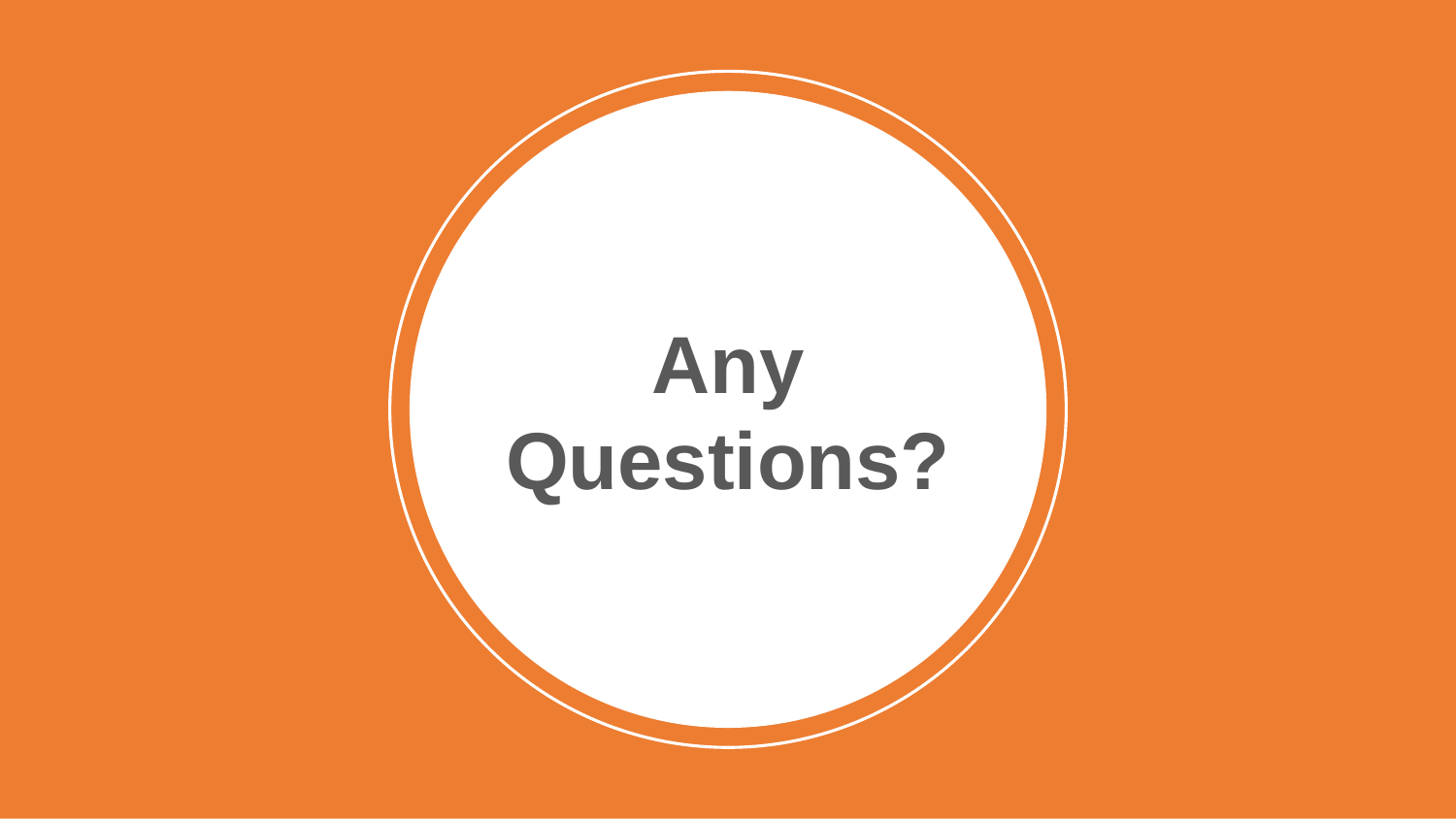

# Any Questions?

## Slide 9
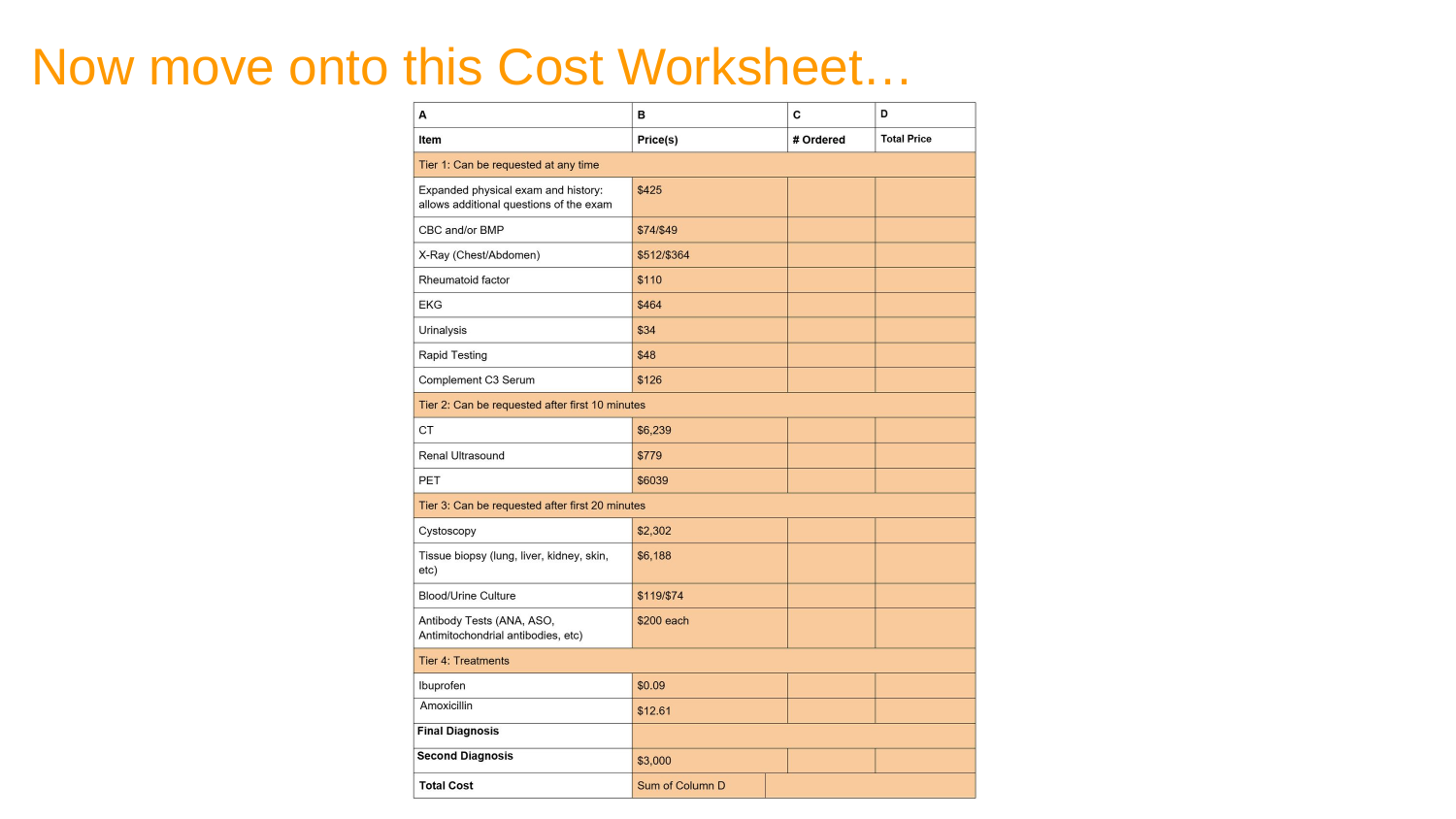

# Now move onto this Cost Worksheet…

## Slide 10
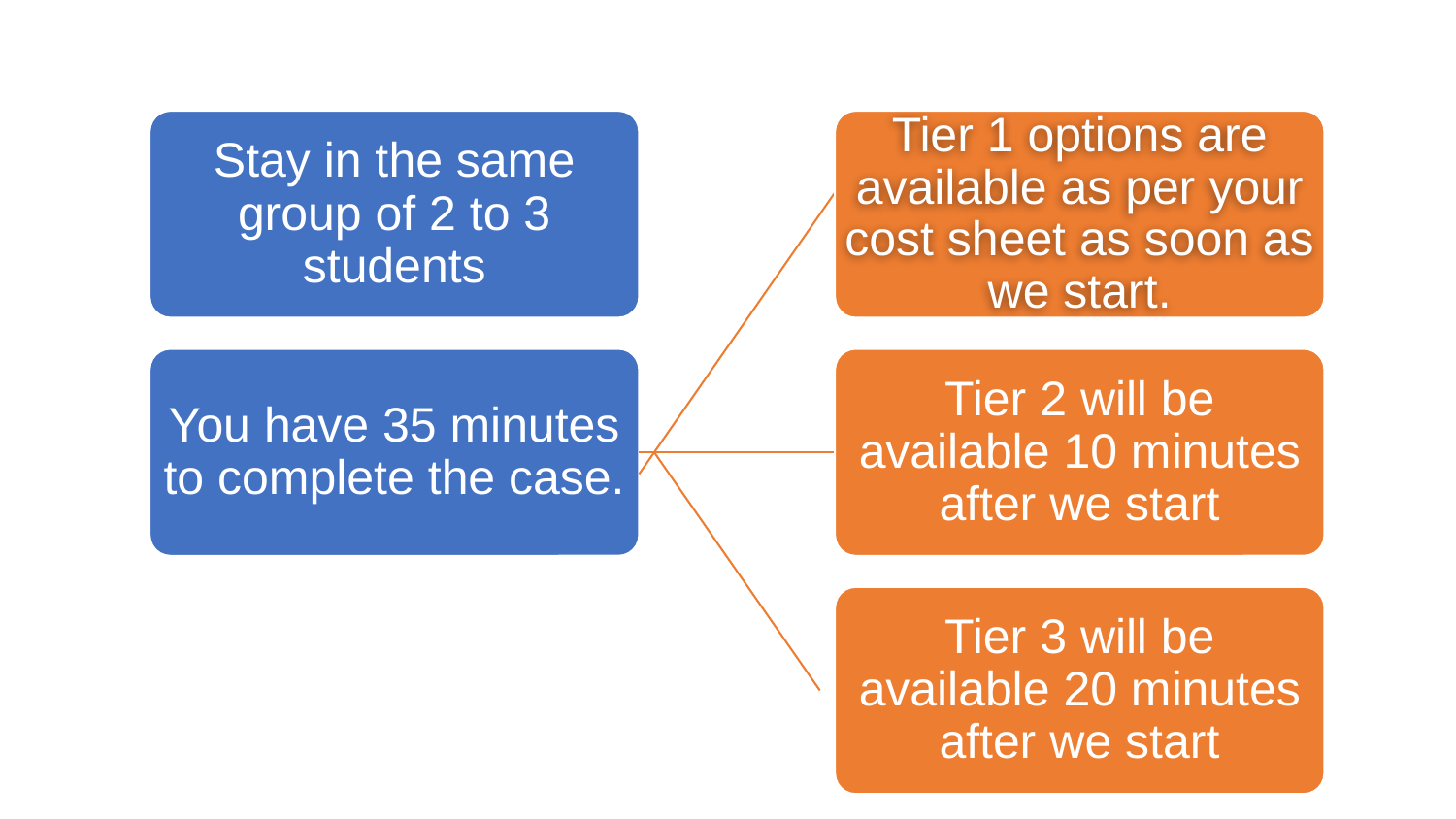

Stay in the same group of 2 to 3 students
Tier 1 options are available as per your cost sheet as soon as we start.
You have 35 minutes to complete the case.
Tier 2 will be available 10 minutes after we start
Tier 3 will be available 20 minutes after we start

## Slide 11
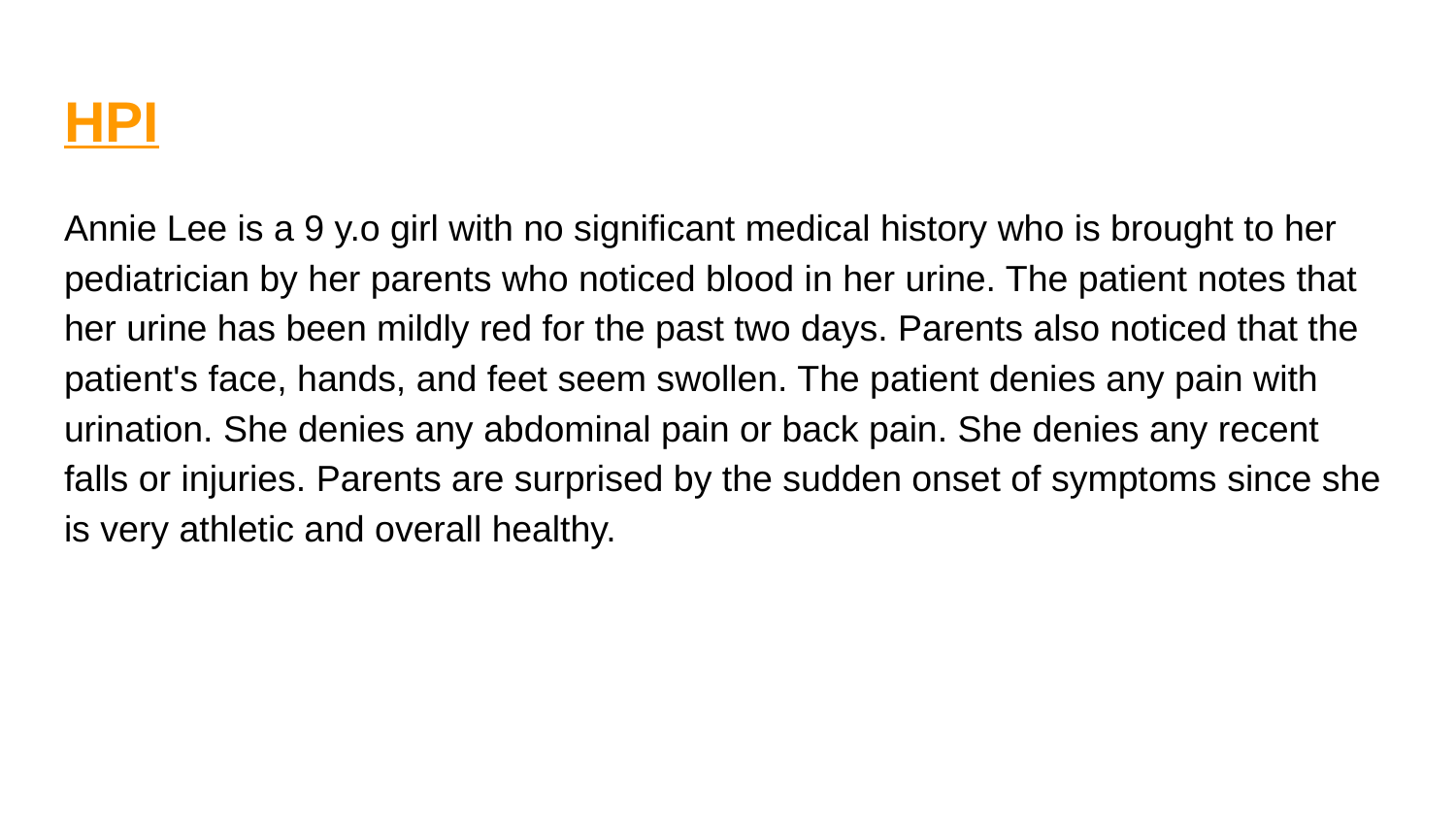

# HPI
Annie Lee is a 9 y.o girl with no significant medical history who is brought to her pediatrician by her parents who noticed blood in her urine. The patient notes that her urine has been mildly red for the past two days. Parents also noticed that the patient's face, hands, and feet seem swollen. The patient denies any pain with urination. She denies any abdominal pain or back pain. She denies any recent falls or injuries. Parents are surprised by the sudden onset of symptoms since she is very athletic and overall healthy.

## Slide 12
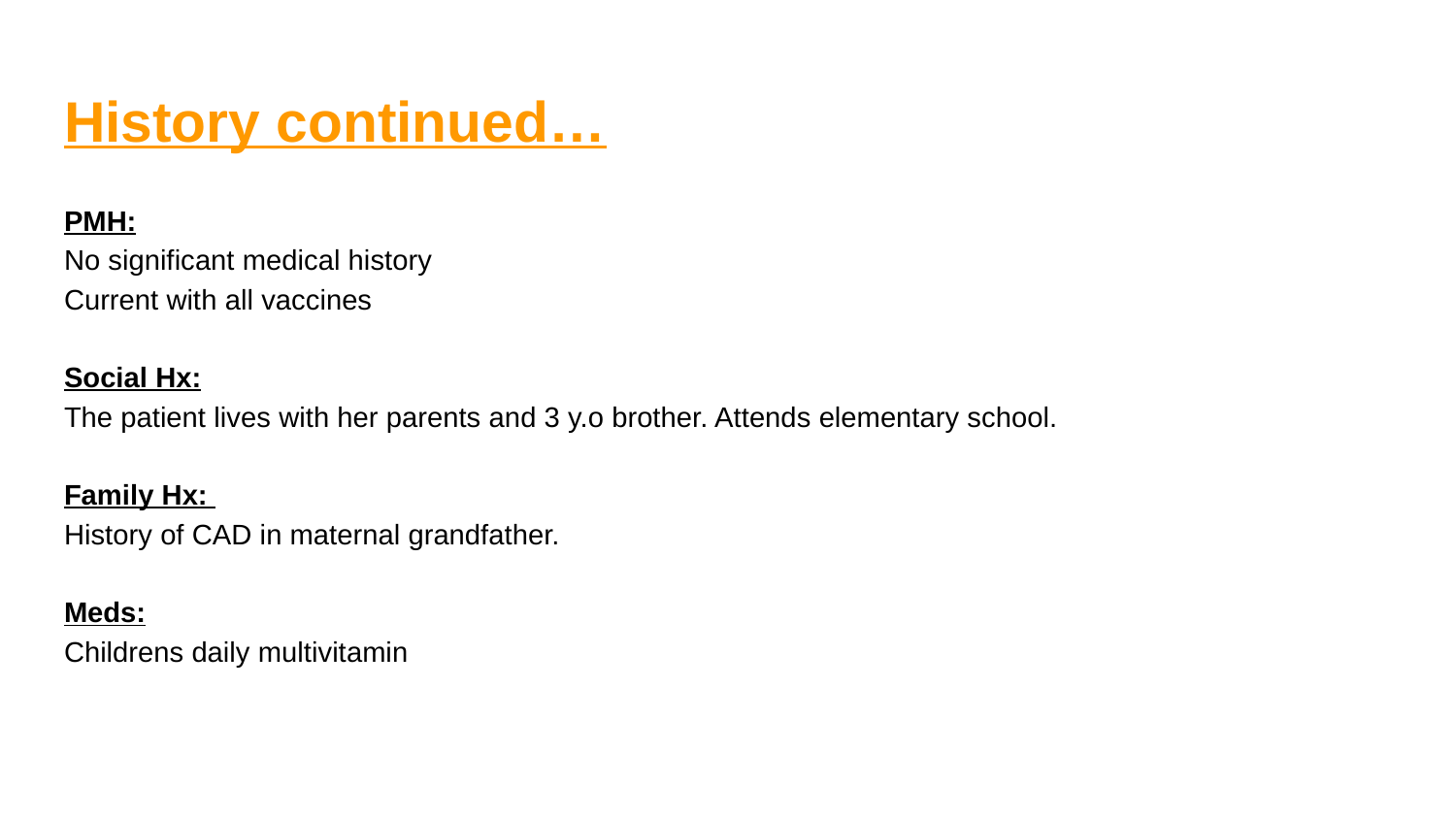

# History continued…
PMH:
No significant medical history
Current with all vaccines
Social Hx:
The patient lives with her parents and 3 y.o brother. Attends elementary school.
Family Hx:
History of CAD in maternal grandfather.
Meds:
Childrens daily multivitamin

## Slide 13
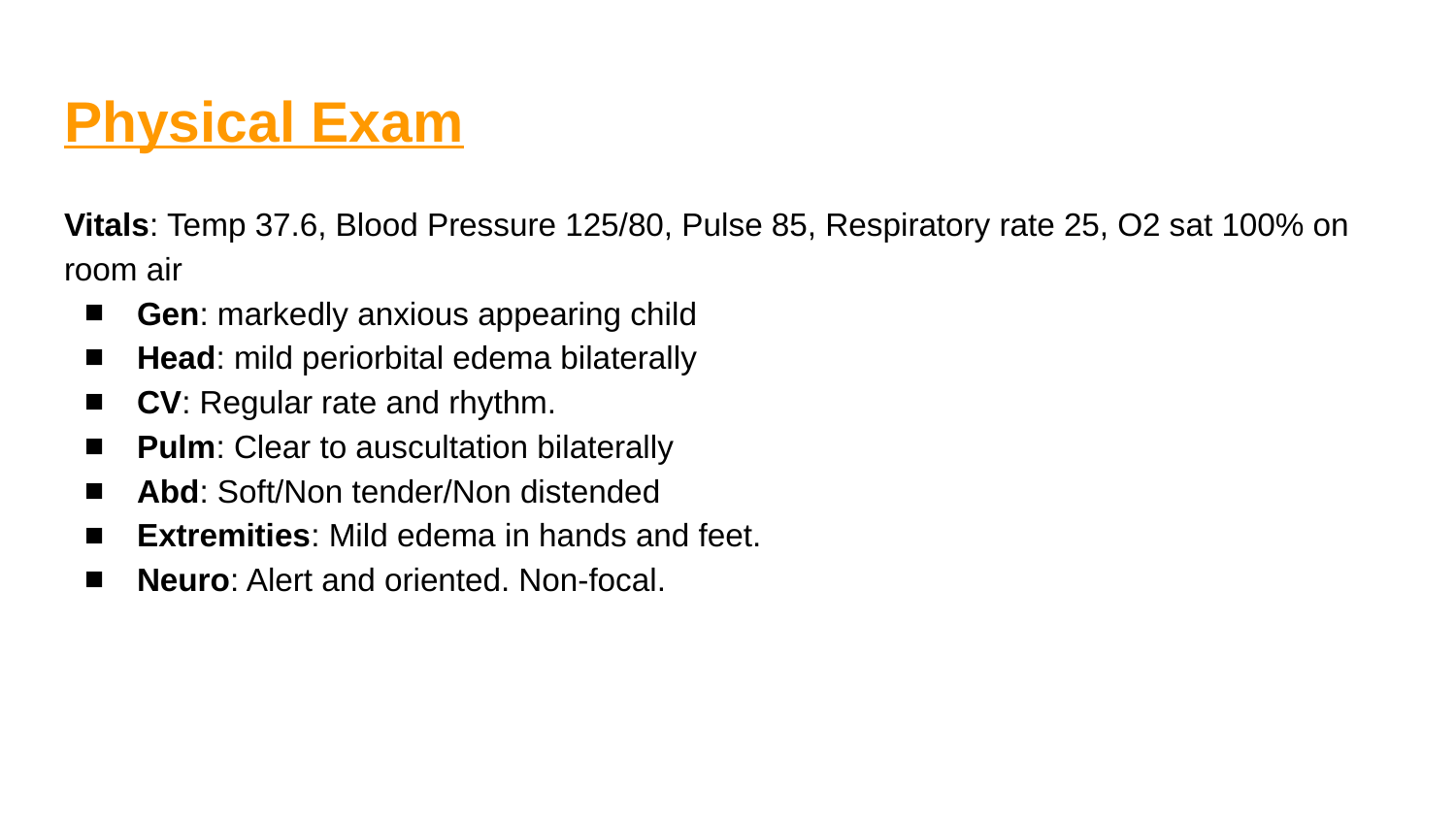

# Physical Exam
Vitals: Temp 37.6, Blood Pressure 125/80, Pulse 85, Respiratory rate 25, O2 sat 100% on room air
Gen: markedly anxious appearing child
Head: mild periorbital edema bilaterally
CV: Regular rate and rhythm.
Pulm: Clear to auscultation bilaterally
Abd: Soft/Non tender/Non distended
Extremities: Mild edema in hands and feet.
Neuro: Alert and oriented. Non-focal.

## Slide 14
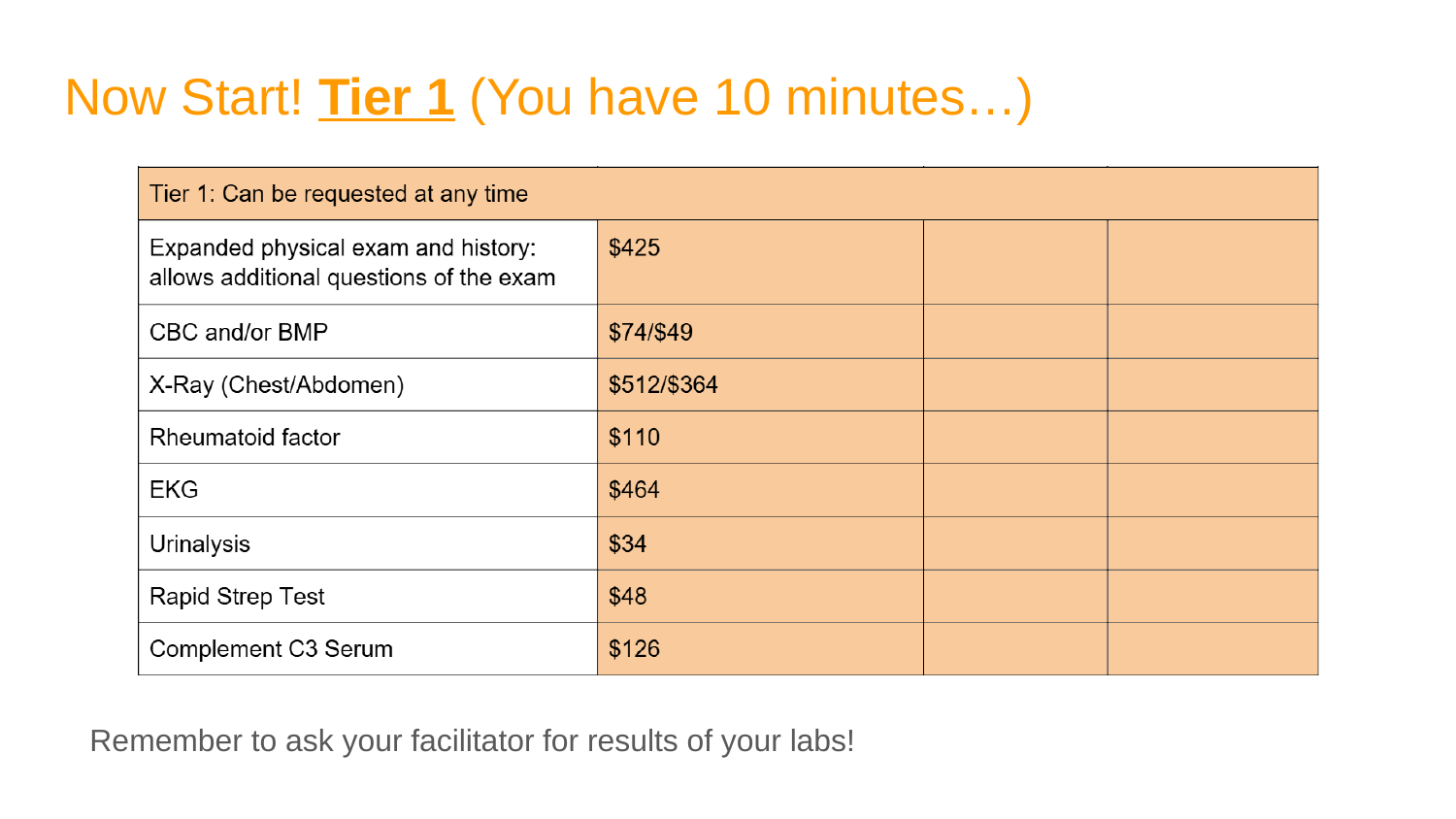

# Now Start! Tier 1 (You have 10 minutes…)
Remember to ask your facilitator for results of your labs!

## Slide 15
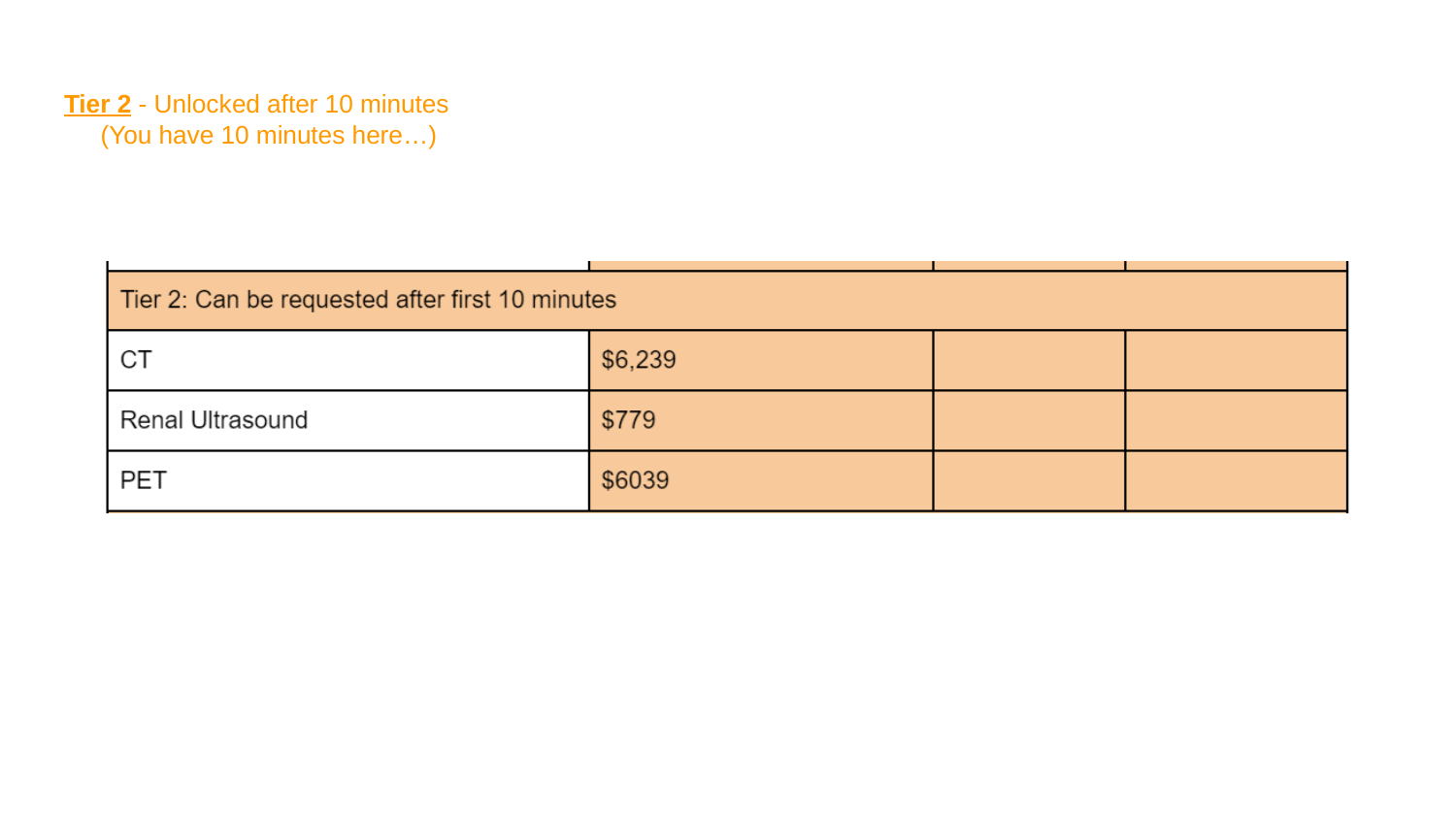

# Tier 2 - Unlocked after 10 minutes
(You have 10 minutes here…)

## Slide 16
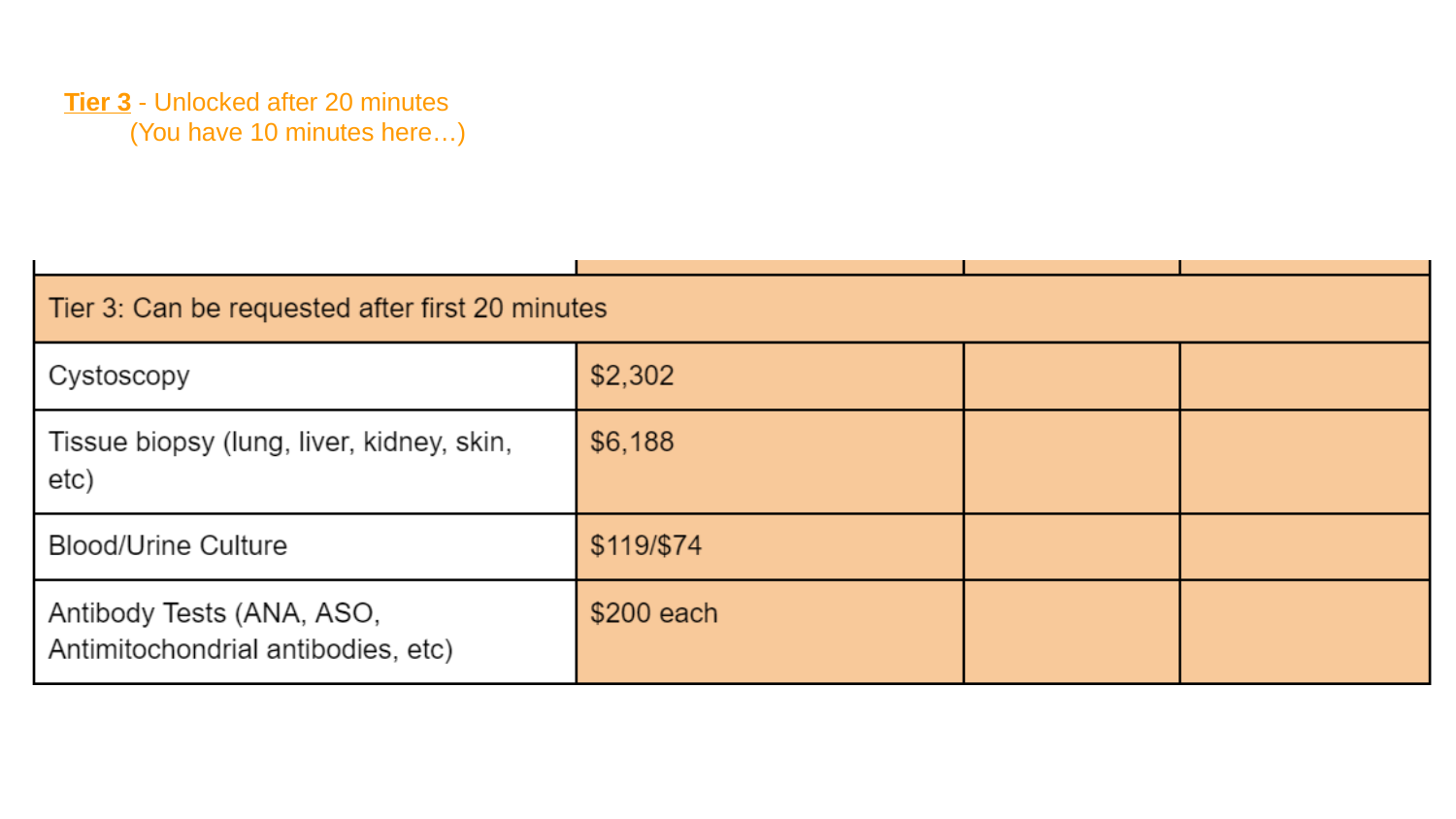

# Tier 3 - Unlocked after 20 minutes
	(You have 10 minutes here…)

## Slide 17
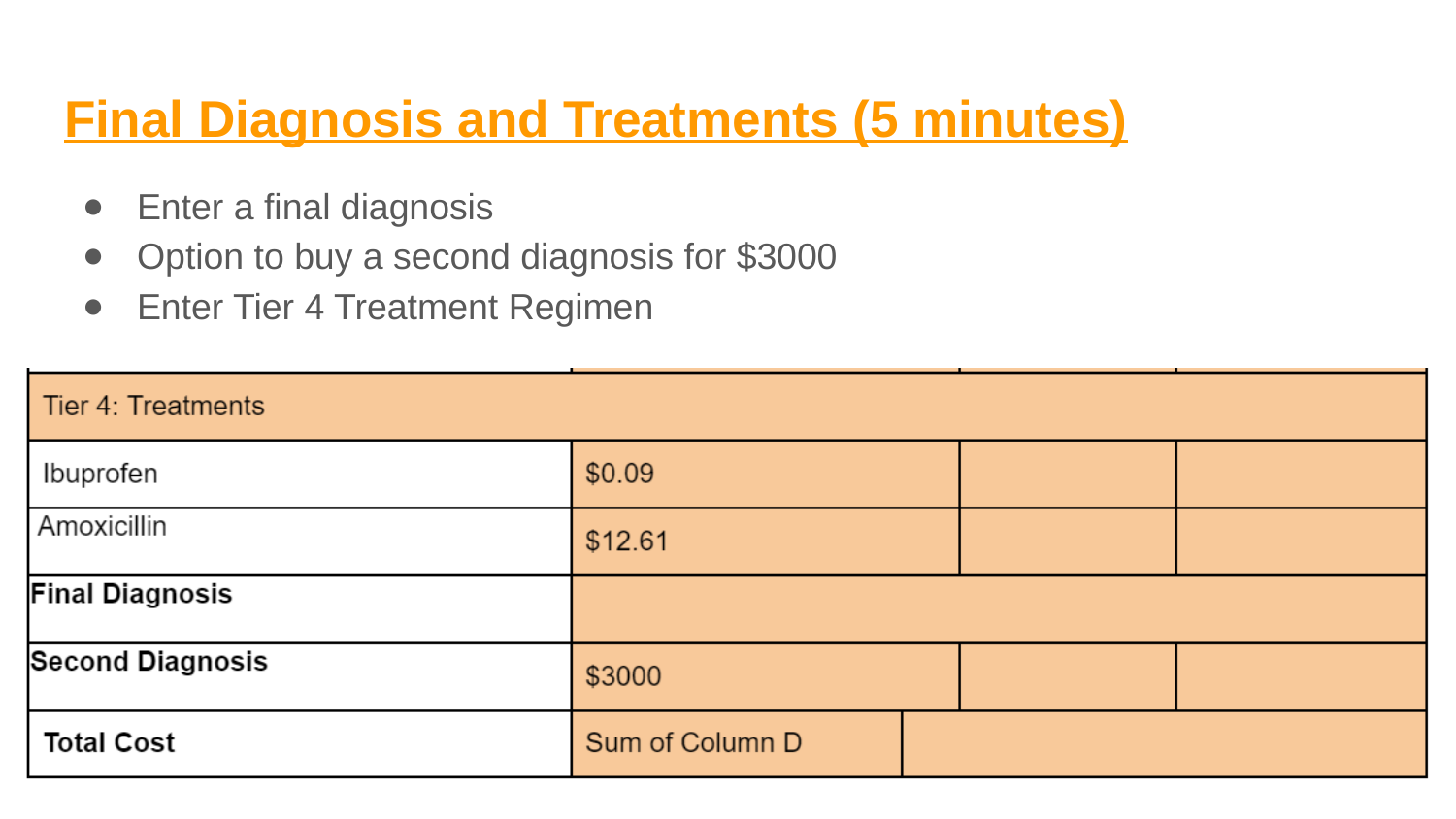

# Final Diagnosis and Treatments (5 minutes)
Enter a final diagnosis
Option to buy a second diagnosis for $3000
Enter Tier 4 Treatment Regimen

## Slide 18
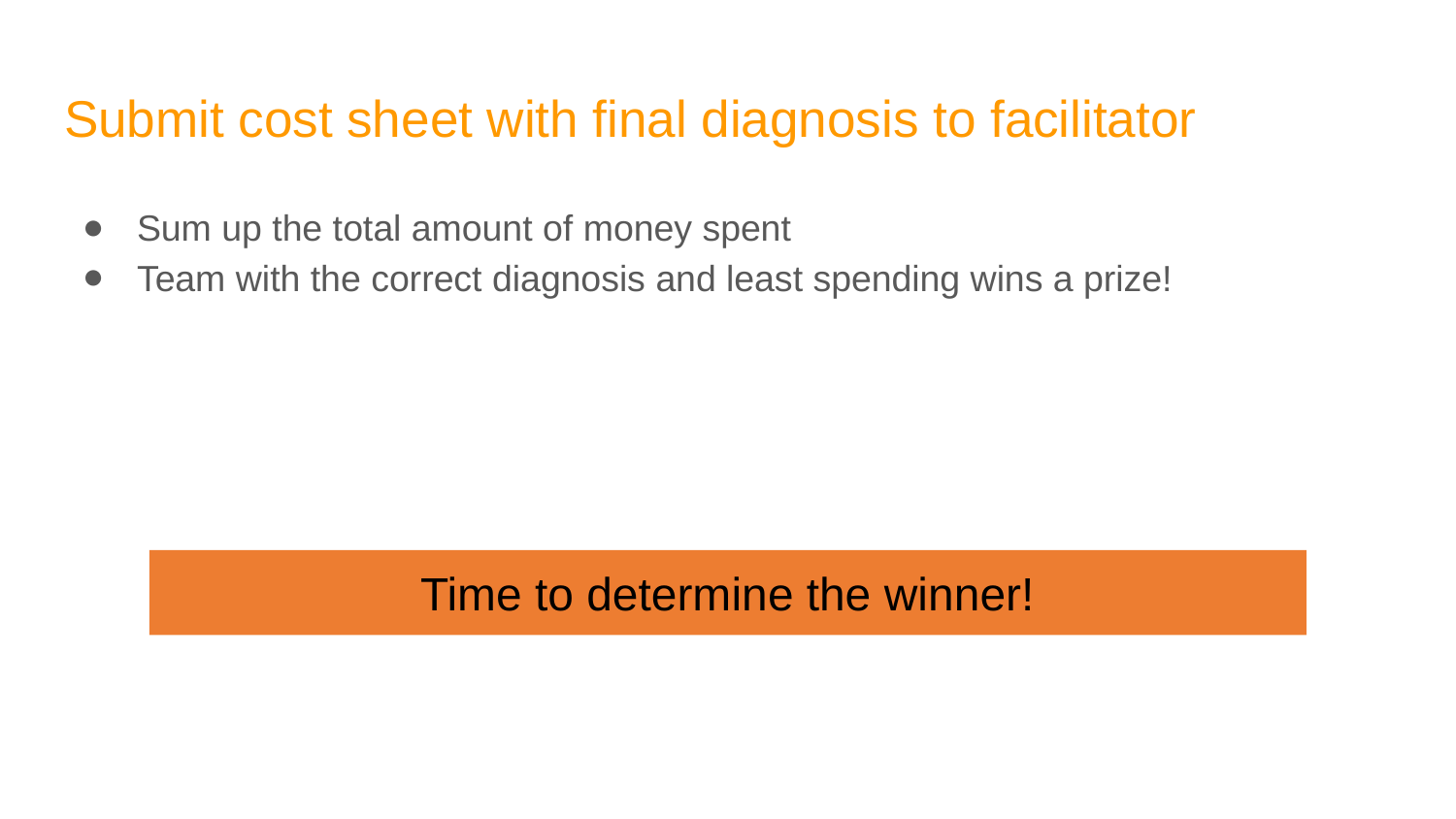

# Submit cost sheet with final diagnosis to facilitator
Sum up the total amount of money spent
Team with the correct diagnosis and least spending wins a prize!
Time to determine the winner!

## Slide 19
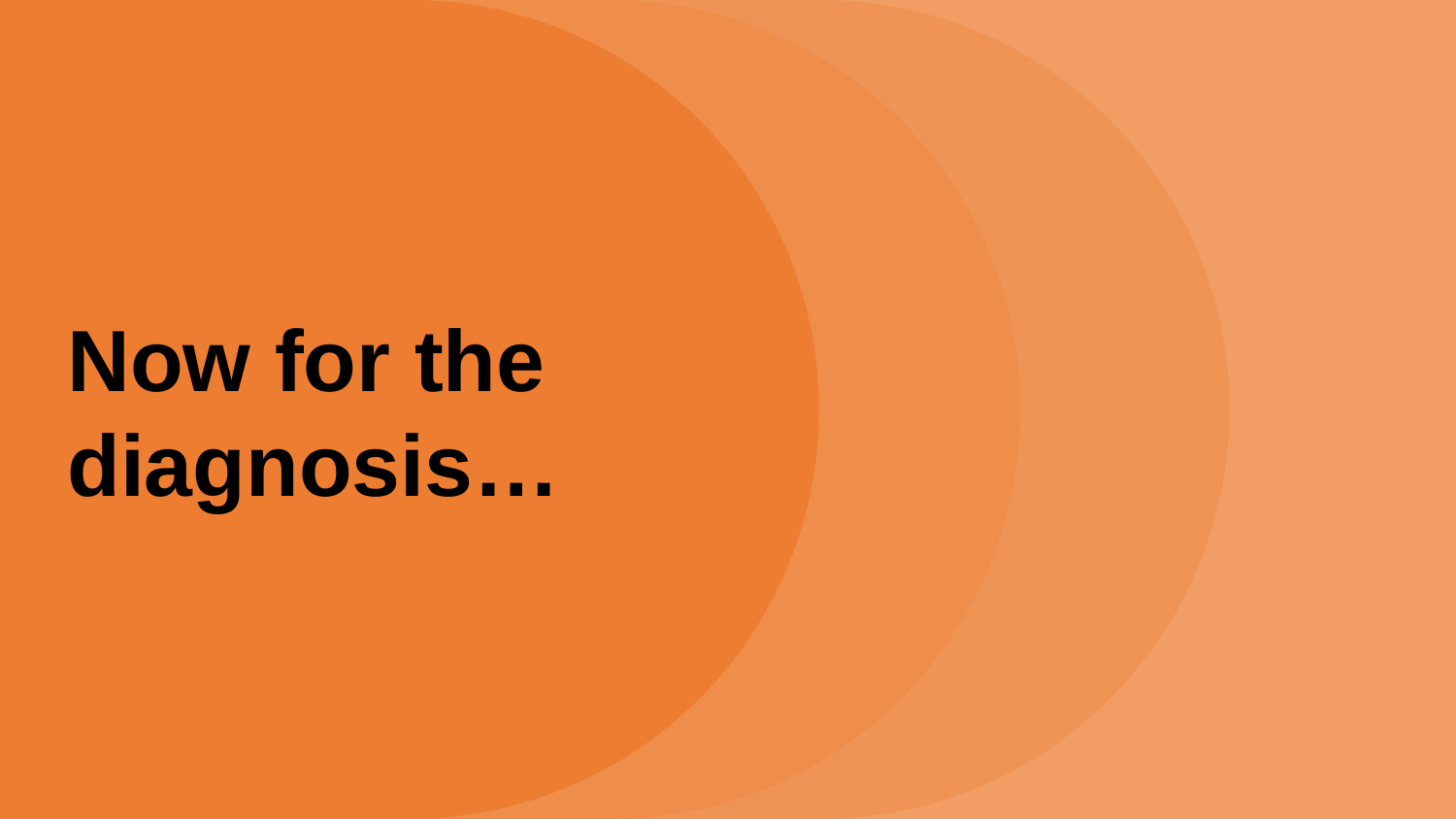

# Now for the diagnosis…

## Slide 20
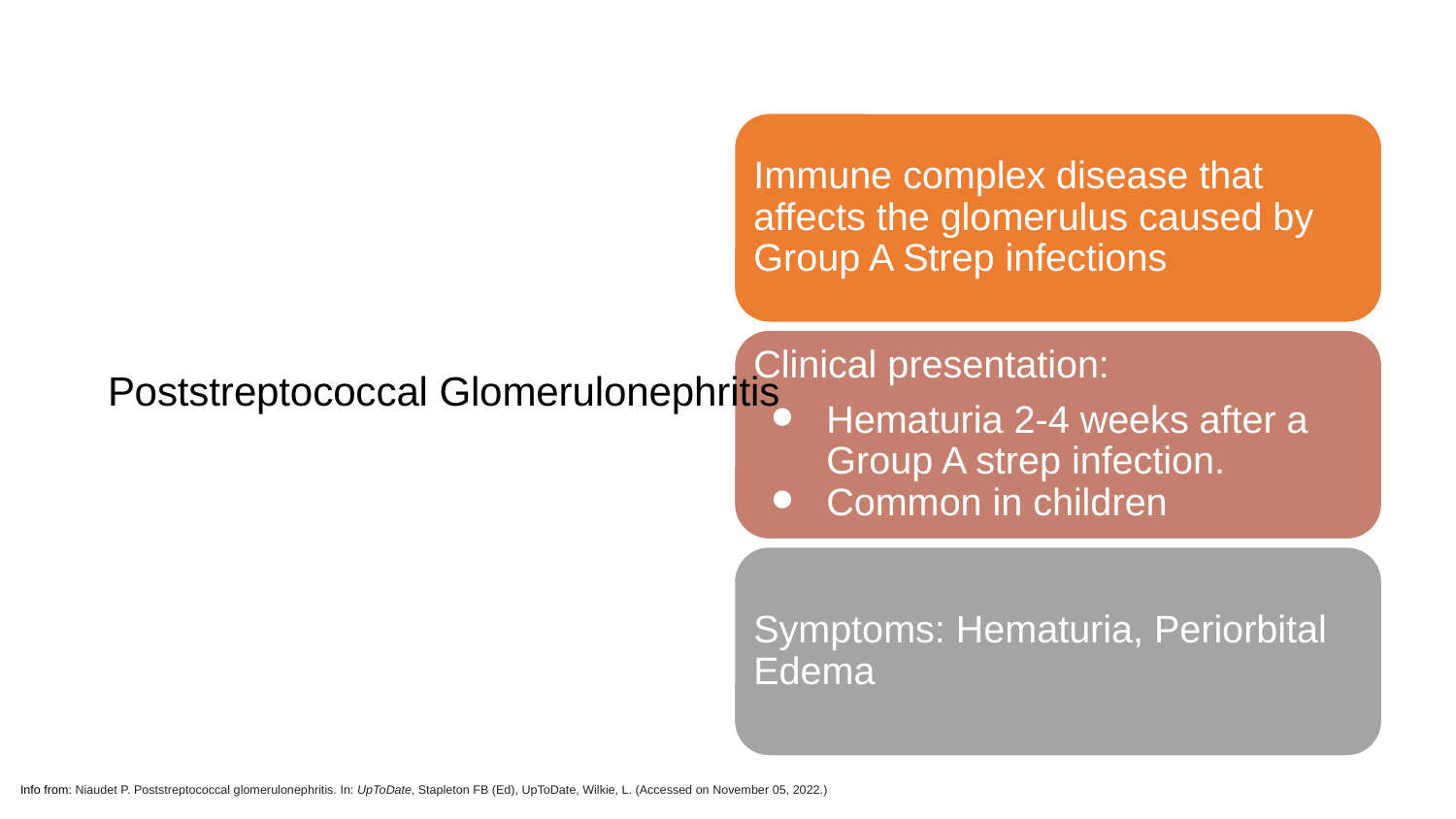

Immune complex disease that affects the glomerulus caused by Group A Strep infections
Clinical presentation:
Hematuria 2-4 weeks after a Group A strep infection.
Common in children
Symptoms: Hematuria, Periorbital Edema
# Poststreptococcal Glomerulonephritis
Info from: Niaudet P. Poststreptococcal glomerulonephritis. In: UpToDate, Stapleton FB (Ed), UpToDate, Wilkie, L. (Accessed on November 05, 2022.)

## Slide 21
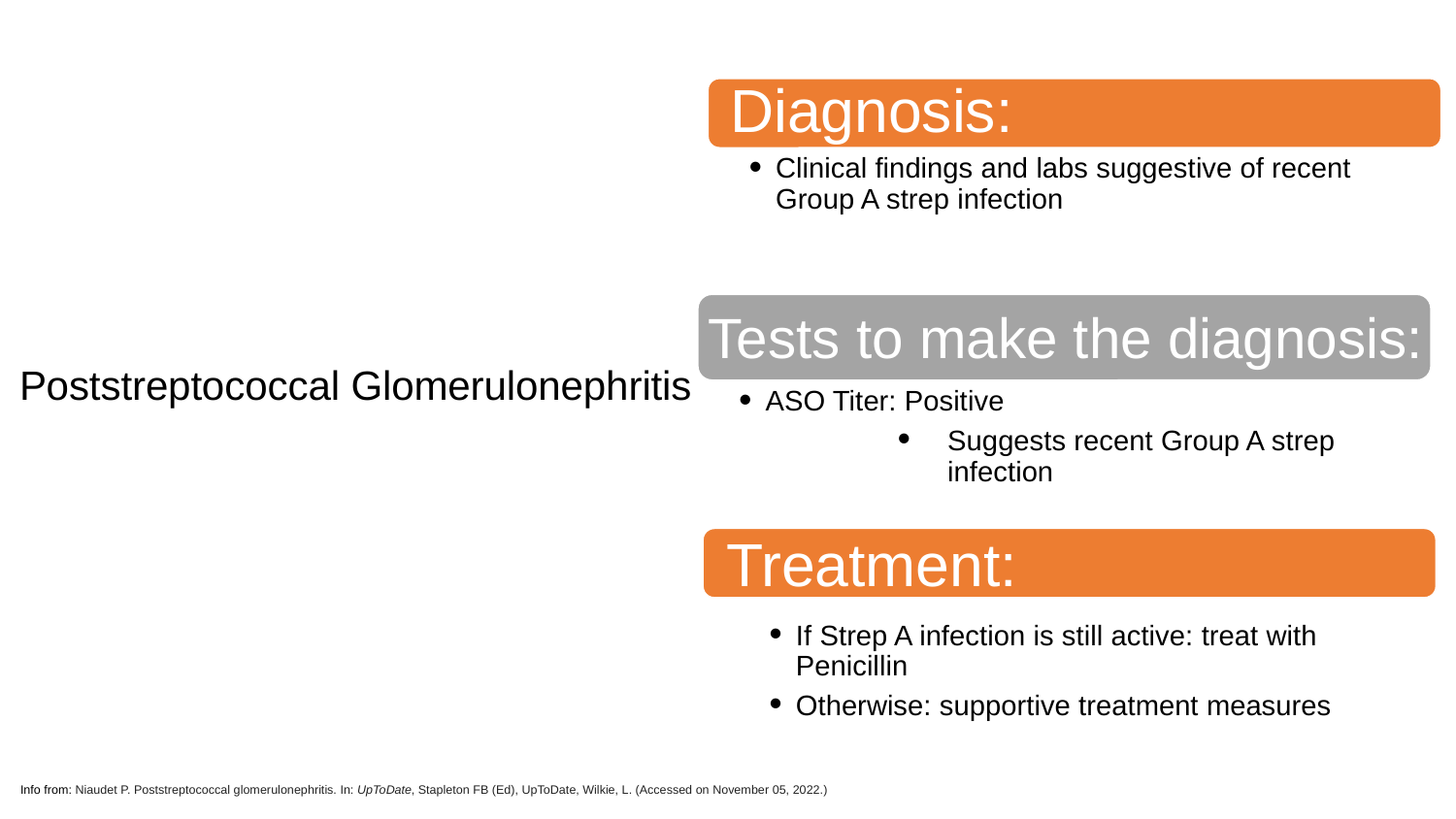

Diagnosis:
Clinical findings and labs suggestive of recent Group A strep infection
Tests to make the diagnosis:
ASO Titer: Positive
Suggests recent Group A strep infection
# Poststreptococcal Glomerulonephritis
Treatment:
If Strep A infection is still active: treat with Penicillin
Otherwise: supportive treatment measures
Info from: Niaudet P. Poststreptococcal glomerulonephritis. In: UpToDate, Stapleton FB (Ed), UpToDate, Wilkie, L. (Accessed on November 05, 2022.)

## Slide 22
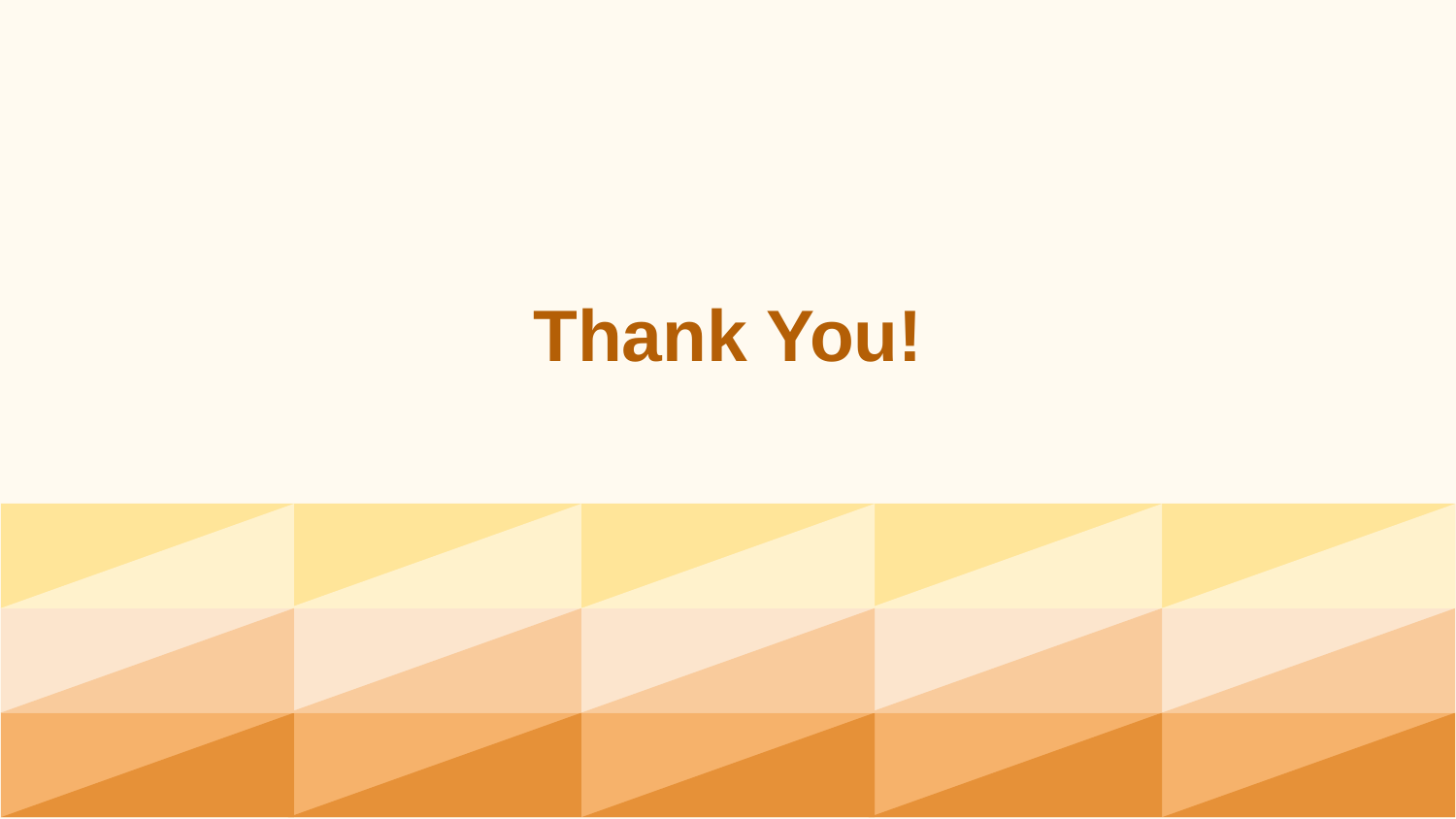

# Thank You!
