# Supplementary material for: Integrating High-Value Cost-Conscious Care into an Existing Medical School Curriculum
Source: MedEdPORTAL. 2025 Jan 28;21:11490. doi: 10.15766/mep_2374-8265.11490 (PMC11772531; doi:10.15766/mep_2374-8265.11490)
Supplement: Supplementary file 1 — Clinical Informatics Pearl 1.docxClinical Informatics Pearl 2.docxClinical Informatics Pearl 3.docxGamified Clinical Skills Lab.pptxCost Worksheet.docxFacilitator Guide.docxPre- and Postsurvey.docx [file mep_2374-8265.11490-s001.zip › F. Facilitator Guide.docx]

**Facilitators Instructions for Gamified CSL**

Curriculum Integration Instruction: A printed or virtual copy of this document should be provided to the facilitator before and during the gamified Clinical Skills Lab for review. It contains detailed instructions on conducting the gamified activity and lab data for facilitators to reference when students request specific labs. This document should not be accessible to students.

# Objectives/Learning Outcomes

By the end of this session students should be able to:

- Develop strategies for high value care decision making
- Describe the role of value-based care in developing treatment plans

# Introduction

The STARS (Students and Trainees Advocating for Resource Stewardship) Gamified CSL is an interactive, competitive simulation to learn and apply concepts of value-based care in a diagnostic reasoning scenario.

# Learning Plan

## Prior to class:

There is no pre-work for this segment of the CSL

## Small group session

The STARS Gamified CSL will occupy the second half of the CSL, i.e. about 45 minutes into the session, leaving about 45 minutes for the competition.

### *Structure*

Students should be divided into teams of 2-3 individuals. In this gamified CSL, students are given the basic information about the case and then have to ask for any other information they need. Each piece of information has associated costs (detailed below) and a time delay (also detailed below) to simulate real life. The goal of the competition is to get the correct diagnosis but at a lower total cost than the opposing team. Each team will track cost using their “cost worksheet”. They will order labs/imaging/procedures etc and the facilitator will provide results for the tests students’ request. The facilitator is provided with a handout with the data for the most likely questions. If students’ ask for something not on the list either make up a normal value or just state the result was unremarkable.

### *Costs*

[Open the “Data for Facilitators” sheet to review the material there as you read this].

Each lab/imaging/procedure’s designated monetary cost based off OHSU’s Charge Master Spreadsheet. The cost for each item is independent. For example BMP costs $49. If the team ordered a BMP and CBC, that would represent two tests and they would be charged for each item ordered for a total of $123. You do not charge them for individual components of a panel, i.e. if they ask for a potassium charge them for a BMP and provide them with the BMP results.

To prevent the team from immediately ordering MRIs or advanced procedures, more complex tests/images/procedures are locked out until a certain amount of time has elapsed. The team is also responsible for tracking their own data results. The students may ask you to go back and look at old slides or repeat the results of tests that have already come back. This is totally fine to do.

***Time***

Every test has an associated time delay to mimic real life where some tests come back fast (like a cbc) and other tests take longer (like an MRI or procedure). To simulate this some basic tests will be available right away but other more advanced tests will not be available until further into the game. Table 1 below provides a detailed description of which types of tests are available throughout the game. Each tier should be visually presented and explained by the facilitator to students using the accompanying PowerPoint slide (Appendix D). Students can also access the full list of items (Appendix E) available for ordering on their cost worksheet. Students will only be able to order items from a specific tier once it opens during the game.

**Table 1.** Timed Tiers and Associated Tests/Treatments

| **Tier** | **Time Available** | **Tests Included** |
| --- | --- | --- |
| Tier 1 | Immediately upon starting game | Expanded physical exam and history: allows additional questions of the exam  CBC and/or BMP  X-Ray (Chest/Abdomen)  Rheumatoid factor  EKG  Urinalysis  Rapid Strep Test  Complement C3 Serum |
| Tier 2 | Available 10 minutes into the game. | CT Abdomen Pelvis  Renal Ultrasound  PET |
| Tier 3 | Available 20 minutes into the game. | Cystoscopy  Invasive tissue biopsy (lung, liver, kidney, skin, etc)  Blood Culture  Urine Culture  Antibody Tests (ANA, ASO, Antimitochondrial antibodies, etc) |
| Tier 4 | Treatment options made available only after diagnosis is made | Ibuprofen  Amoxicillin |

### *Facilitator Role*

As above, the role of the facilitator in the room is to mostly provide data from the “Data for Facilitators” (found below) at the appropriate time intervals and help make sure the team is tracking their costs. The facilitator should not attempt to guide the discussion. The purpose of this is for students to order many tests, shoot around in the dark, and perhaps understand what it means to do some non-directed wasted spending. Facilitators have the additional role of keeping track of time and informing students when the next “Tier” of information is available.

### *Game Play and Cost Worksheet*

The cost-worksheet should be managed by the team and not the facilitator. There are three Tiers of testing that will become available during the duration of the time.

- Game starts when teams go into separate rooms after they have heard the HPI.
- Tier 1: Available immediately upon starting the game. Each test has a monetary cost.
- Tier 2: Available 10 minutes into the game. Each test has a monetary cost.
- Tier 3: Available 20 minutes into the game. Each test has a monetary cost.
- Game ends after 35 minutes

It is important that when teams decide to “order a test” that they commit to ordering and write it down on their “cost worksheet”. Nothing is ordered until it is written down. At the end of the game, the students should tally up the total cost based on tests ordered and sum everything up at the bottom of column C.

### *Odds and Ends*

As above, the way a team wins is to get the correct diagnosis at the lowest total cost. In the event neither team gets the diagnosis then the team with the diagnosis on their differential wins. If both teams have it on their differential than the team that spent the least money wins. Of note, there is the option to buy a second diagnosis slot which effectively gives a team two guesses at the final diagnosis though this is an intentionally expensive option.

**EXAMPLE TIMELINE FOR THE STARS GAMIFIED CSL**

An example timeline for the STARS Gamified CSL is detailed in Table 2 below:

**Table 2.** Timeline of Gamified CSL

| **Time (min)** | **Events and Tasks** |
| --- | --- |
| 0 | Review the intro slides that explain the case competition & review the HPI |
| 10 | Separate into teams and the case officially begins. Tier 1 tests are available immediately |
| 20 | Tier 2 tests are available. |
| 30 | Tier 3 tests are available. |
| 45 | Deadline to submit final diagnosis. Treatment Tier 4 is open when diagnosis is submitted. |
| 50 | Total up money spent. Winners of the competition receive a prize bag containing goodies. |
| 60 | Review education slides & debrief. |

**DATA FOR FACILITATORS**

**They must ask for this information. DO NOT JUST READ THE INFORMATION BELOW. Only give information that they directly query for. If not listed below, presume the answer is negative**

**Working Case (35 minutes) - Post-streptococcal glomerulonephritis**

Brief teaching points from Up to Date^1^:

| **Diagnosis of PSGN:** PSGN is typically identified through clinical signs of acute nephritis and evidence of a recent group A beta-hemolytic streptococcal (GAS) infection.  **Clinical signs of acute nephritis:** Includes hematuria (with or without red blood cell casts), varying levels of proteinuria, swelling (edema), reduced urine output (oliguria), and high blood pressure (hypertension).  **Evidence of recent GAS infection:** Confirmed through a positive throat or skin culture or serologic tests such as the anti-streptolysin (ASO) or streptozyme test.  **Treatment:**  **Antibiotic therapy:** If the streptococcal infection is still present at diagnosis, penicillin should be administered (or erythromycin for those allergic to penicillin). Although not certain, early treatment of the streptococcal infection may help prevent or lessen the severity of glomerulonephritis.  **Supportive care:** There is no specific treatment for PSGN. Management focuses on addressing the symptoms, particularly those related to volume overload, such as hypertension and, less commonly, pulmonary edema. General measures include restricting sodium and water intake and using loop diuretics. |
| --- |

**Therefore, the most optimal solution: Urinalysis and ASO Test. Supportive treatment measures.**

**They must ask for this information. DO NOT JUST READ THE INFORMATION BELOW. Only give information that they directly query for. If not listed below, presume the answer is negative**

**EXPANDED HISTORY**

**Previous illness:** Parents note that the patient had a cold two weeks ago that presented with fever, sore throat, and rash. However, they note that this resolved after a few days.

**Travel history:** No recent travel history

**Sick Contacts:** Younger brother had similar cold two weeks ago. No known exposures to ticks or pets.

**Drug/Alcohol History:** Never smoked, drank alcohol or used illicit substances

**EXPANDED PHYSICAL EXAM**

**Neuro:** completely normal , no focal weakness, no sensory deficits, no cranial nerve deficits, reflexes are within normal limits

**Abdomen:** no hepatosplenomegaly detected.

**Back Exam:** CVA region tender to percussion

**Derm:** no rashes

**IMAGING/PROCEDURES**

**If not below presume the result is non-diagnostic or negative.**

**EKG**: Normal sinus rhythm without acute ST or T wave changes

**CXR**: Unremarkable

**CT A/P**: Unremarkable

**CT Thorax**: Unremarkable

**CT Guided Kidney Biopsy**: if obtained, a pathology report will be returned. However, all tests that are to be run on it must be requested individually (see Pathology section for individual results)

**Cystoscopy:** No abnormalities

**PATHOLOGY**

**CT guided biopsy pathology results:**

- **Renal Biopsy:**
  - Light microscopy of biopsy shows a diffuse proliferative and exudative glomerulonephritis with prominent endocapillary proliferation and numerous neutrophils
  - Immunofluorescence (IF) microscopy reveals a characteristic pattern of deposits of C3 and immunoglobulin G (IgG) distributed in a diffuse granular pattern within the mesangium and glomerular capillary walls
- All other biopsies are normal.

**LABS**

**Routine Labs**

**BMP:**: 134 (Na: 135-145), 3.3 (K: 3.5-5.1), 99 (Cl: 94-106), 24.6 (CO2: 20-29), 31 (BUN: 7-25), 1.7 (Cr: 0.6-1.3), 158 (Glucose: 60-100)

**CBC**: WBC 4.6, Hgb 12.1, Hct 36.1, Plt 250.

**UA**: dark red colored urine with hematuria, RBC casts and mild proteinuria.

**Rapid Test:** Negative

**Complement C3**: Low - 57 mg/dl (normal: 88 to 201 mg/dl)

**Rheumatologic**

**SPEP/UPEP:** negative

**RF:** negative

**ANA:** negative

**Other rheumatologic serologies (anti-smith, anti-jo, anti-DS DNA etc):** ASO positive, all other negative

**Lyme titer:** undetectable

**Microbiology/Infectious Disease**

**Blood cultures:** no growth at five days

**Urine culture:** no growth

**References**

Niaudet P. Poststreptococcal glomerulonephritis. In: Stapleton FB, ed. *UpToDate*. Wilkie L, ed. UpToDate. Accessed November 5, 2022. <https://www.uptodate.com>
