# Supplementary material for: Integrating High-Value Cost-Conscious Care into an Existing Medical School Curriculum
Source: MedEdPORTAL. 2025 Jan 28;21:11490. doi: 10.15766/mep_2374-8265.11490 (PMC11772531; doi:10.15766/mep_2374-8265.11490)
Supplement: Supplementary file 1 — Clinical Informatics Pearl 1.docxClinical Informatics Pearl 2.docxClinical Informatics Pearl 3.docxGamified Clinical Skills Lab.pptxCost Worksheet.docxFacilitator Guide.docxPre- and Postsurvey.docx [file mep_2374-8265.11490-s001.zip › E. Cost Worksheet.docx]

**STARS Gamified CSL Clinical Case**

Curriculum Integration Instruction: Medical students should receive a printed or virtual copy of this sheet to accompany the gamified Clinical Skills Lab activity. They can reference the clinical scenario and cost worksheet included in this document.

**HPI:**

Annie Lee is a 9 y.o girl with no significant medical history who is brought to her pediatrician by her parents who noticed blood in her urine. The patient notes that her urine has been mildly red for the past two days. Parents also noticed that the patient's face, hands, and feet seem swollen. The patient denies any pain with urination. She denies any abdominal pain or back pain. She denies any recent falls or injuries. Parents are surprised by the sudden onset of symptoms since she is very athletic and overall healthy.

**PMH:**

No significant medical history

Current with all vaccines

**Social Hx:**

The patient lives with her parents and 3 y.o brother. Attends elementary school.

**Family Hx:**

History of CAD in maternal grandfather.

**Exam:** Vitals: Temp 37.6, Blood Pressure 125/80, Pulse 85, Respiratory rate 25, O2 sat 100% on room air

- Gen: markedly anxious appearing child
- Head: mild periorbital edema bilaterally
- CV: Regular rate and rhythm.
- Pulm: Clear to auscultation bilaterally
- Abd: Soft/Non tender/Non distended
- Extremities: Mild edema in hands and feet.
- Neuro: Alert and oriented. Non-focal.

**Meds:**

Childrens daily multivitamin

| **Item** | **Price(s)** | | **# Ordered** | **Total Price** |
| --- | --- | --- | --- | --- |
| Tier 1: Can be requested at any time | | | | |
| Expanded physical exam and history: allows additional questions of the exam | $425 | |  |  |
| CBC and/or BMP | $74/$49 | |  |  |
| X-Ray (Chest/Abdomen) | $512/$364 | |  |  |
| Rheumatoid factor | $110 | |  |  |
| EKG | $464 | |  |  |
| Urinalysis | $34 | |  |  |
| Rapid Strep Test | $48 | |  |  |
| Complement C3 Serum | $126 | |  |  |
| Tier 2: Can be requested after first 10 minutes | | | | |
| CT | $6,239 | |  |  |
| Renal Ultrasound | $779 | |  |  |
| PET | $6039 | |  |  |
| Tier 3: Can be requested after first 20 minutes | | | | |
| Cystoscopy | $2,302 | |  |  |
| Tissue biopsy (lung, liver, kidney, skin, etc) | $6,188 | |  |  |
| Blood/Urine Culture | $119/$74 | |  |  |
| Antibody Tests (ANA, ASO, Antimitochondrial antibodies, etc) | $200 each | |  |  |
| Tier 4: Treatments | | | | |
| Ibuprofen | $0.09 | |  |  |
| Amoxicillin | $12.61 | |  |  |
| **Final Diagnosis** |  | | | |
| **Second Diagnosis** | $3,000 | |  |  |
| **Total Cost** | Sum of Column D |  | | |
