# Supplementary material for: Integrating High-Value Cost-Conscious Care into an Existing Medical School Curriculum
Source: MedEdPORTAL. 2025 Jan 28;21:11490. doi: 10.15766/mep_2374-8265.11490 (PMC11772531; doi:10.15766/mep_2374-8265.11490)
Supplement: Supplementary file 1 — Clinical Informatics Pearl 1.docxClinical Informatics Pearl 2.docxClinical Informatics Pearl 3.docxGamified Clinical Skills Lab.pptxCost Worksheet.docxFacilitator Guide.docxPre- and Postsurvey.docx [file mep_2374-8265.11490-s001.zip › G. Pre- and Postsurvey.docx]

Curriculum Integration Instruction: The pre-survey should be administered to students before the first Clinical Informatics Pearl (Appendix A) to establish baseline knowledge and attitudes. The “Satisfaction Questions” at the end of this document should be excluded from the pre-survey but included in the post-survey. The post-survey should be conducted after the final activity, the Gamified Clinical Skills Lab.

You will be asked to complete a survey about high-value cost-conscious care prior to completing todays Clinical Pearl Activity. This survey is a mandatory part of the curriculum and must be completed as part of getting credit for this Pearl activity.

1. **Please enter your name: __________________________**

1. **Knowledge Questions:**
   1. Which imaging/screening modalities should you perform for uncomplicated headaches on a pediatric patient with symptoms for longer than 5 months:
      1. MRI
      2. CT scan
      3. Lumbar Puncture
      4. None
   2. Which of the following resources can you use to check lab and medication costs at [Insert Institution] (select all that apply):
      1. GoodRx [can substitute with any medication cost resource]
      2. [University cost resource] Excel Sheet
      3. [EHR]
      4. UpToDate
   3. In [insert EHR], which of the following places can you check to see if labs/imaging have been previously ordered? (select all that apply)
      1. Chart Review
      2. Results Review
      3. Snapshot
      4. History

1. **Attitudes Questions: Indicate how much you agree or disagree with each of the following statements. (Ranking scale: Strong Disagree; Disagree; Agree; Strongly Agree):**

From MHAQ^1^

|  | Strongly disagree | Disagree | Agree | Strongly Agree |
| --- | --- | --- | --- | --- |
| 1. Physicians should try not to think about the cost to the health care system when making treatment decisions. | ○ | ○ | ○ | ○ |
| 2. Physicians should be aware of the costs of the tests or treatments they recommend. | ○ | ○ | ○ | ○ |
| 3. Physicians should talk to patients about the costs of care when discussing treatment options. | ○ | ○ | ○ | ○ |
| 4. Physicians should change their clinical practices (e.g., ordering, prescribing) if the costs of care they provide is higher than colleagues who care for similar patients. | ○ | ○ | ○ | ○ |
| 5. Physician clinical practices (e.g., ordering, prescribing) are key drivers of high health care costs. | ○ | ○ | ○ | ○ |
| 6. Costs to society should be important in physician decisions to use or not to use an intervention. | ○ | ○ | ○ | ○ |
| 7. It is unfair to ask physicians to be cost conscious and still keep the welfare of their patients foremost in their minds. | ○ | ○ | ○ | ○ |
| 8. Cost-effectiveness data should be used to determine what treatments are offered to patients. | ○ | ○ | ○ | ○ |
| 9. Trying to contain costs is the responsibility of every physician. | ○ | ○ | ○ | ○ |
| 10. Physicians should discuss cost efficiency of care with their patients. | ○ | ○ | ○ | ○ |

**3. Effectiveness question**

Please rate your comfort accessing resources to provide cost-effective care:

- - 1. Very uncomfortable
    2. Uncomfortable
    3. Neutral
    4. Comfortable
    5. Very comfortable

**Satisfaction Question (only in post-survey): The following questions address the curriculum intervention you participated in which included three informatics pearls on high-value, cost-conscious care (HVCCC) resources and the gamified HVCCC CSL.**

1. Please rate the effectiveness of this curriculum in promoting cost-effective care: a. Ineffective
   1. Mildly effective
   2. Somewhat effective
   3. Effective
   4. Very effective

1. How likely are you to use the resources/skills provided in this curriculum to practice cost effective care during your clinical rotations:
   1. Very unlikely
   2. Unlikely
   3. Somewhat likely
   4. Likely
   5. Very likely

**References**

1. Mordang, S., Könings, K. D., Leep Hunderfund, A. N., Paulus, A., Smeenk, F., & Stassen, L. (2020). A new instrument to measure high value, cost-conscious care attitudes among healthcare stakeholders: development of the MHAQ. *BMC health services research*, *20*(1), 156. https://doi.org/10.1186/s12913-020-4979-z
