# Supplementary material for: Integrating High-Value Cost-Conscious Care into an Existing Medical School Curriculum
Source: MedEdPORTAL. 2025 Jan 28;21:11490. doi: 10.15766/mep_2374-8265.11490 (PMC11772531; doi:10.15766/mep_2374-8265.11490)
Supplement: Supplementary file 1 — Clinical Informatics Pearl 1.docxClinical Informatics Pearl 2.docxClinical Informatics Pearl 3.docxGamified Clinical Skills Lab.pptxCost Worksheet.docxFacilitator Guide.docxPre- and Postsurvey.docx [file mep_2374-8265.11490-s001.zip › _Educational Summary Report_11490.pdf]

MedEdPORTAL® The AAMC Journal of Teaching and Learning Resources

Original Publication Open Access

Integrating High-Value Cost-Conscious Care into an Existing Medical School Curriculum

Sruthi Eapen*,†, Aylmer Tan†, Paul Gorman, MD, Gretchen Scholl, MC, Andrea Smeraglio, MD

*Corresponding author: eapen@ohsu.edu
†Co-primary author

Abstract

Introduction: High-value cost-conscious care (HVCCC) education has been shown to reduce wasteful health care spending. Incorporating HVCCC into a medical school curriculum can be challenging due to limited curricular time. We explored the feasibility of medical students creating HVCCC peer education within existing platforms at a single urban academic medical school. We reasoned that curricular changes could improve student knowledge, attitudes, and competency with HVCCC within 2 hours and 25 minutes of curricular time. **Methods:** First-year medical student attitudes and understanding regarding HVCCC were evaluated via a survey before and after the delivery of a mixed asynchronous and in-person HVCCC curriculum created by two medical student peers. The curricula comprised three spaced asynchronous online sessions targeting HVCCC skill development followed by a gamified 90-minute clinical skills lab where students competed to determine the correct diagnosis at the lowest cost. **Results:** One hundred and twenty-three medical students (out of 145 first-year medical students) completed the presurvey and indicated willingness to participate in the educational innovation, and 54 completed both surveys. Forty-two percent of students agreed/strongly agreed that the curriculum was effective/strongly effective at promoting cost-effective care. Sixty-five percent of students agreed they would likely use these resources during their clinical rotations. Comfort accessing HVCCC resources improved from 4% precurriculum to 41% postcurriculum. There was no significant difference in HVCCC knowledge pre- and postsurvey. **Discussion:** This educational innovation demonstrated the feasibility of a peer-developed HVCCC curriculum in preclinical education that minimally impacted curricular time and improved student comfort in accessing cost-effective resources.

Keywords
Games, Game-Based Learning, High-Value Care/Cost-Conscious Care, High-Value Cost-Conscious Care

Educational Objectives

By the end of this activity, learners will be able to:

1. Use widely available online tools that aid with high-value care decision-making.
2. Describe the role of value-based care in developing treatment plans.

Introduction

The United States has the highest health care costs amongst developed nations with approximately 25% being considered waste.[1] Physicians play a significant role in this waste when they order low-value tests, imaging, and interventions.[2] Despite the role physicians play in health care costs, medical students continue to graduate from medical school feeling inadequately trained to provide high-value cost-conscious care (HVCCC). The 2022 AAMC Medical School Graduation Questionnaire (GQ) found that only 41.6% of students strongly agreed that they have the skills to apply the principles of high-value care in residency.[3] This is one of the lowest-scoring domains on the GQ residency preparedness question suggesting that there remains significant room for improvement in HVCCC training.[3] Furthermore, experts have called on integrating HVCCC concepts earlier into medical education.[4]

In 2015 a grassroots organization called STARS (Students and Trainees Advocating for Resource Stewardship) was launched to improve HVCCC integration into medical student education. STARS promotes HVCCC through student-led initiatives to

Citation:
Eapen S, Tan A, Gorman P, Scholl G, Smeraglio A. Integrating high-value cost-conscious care into an existing medical school curriculum. *MedEdPORTAL*. 2025;21:11490.
https://doi.org/10.15766/mep_2374-8265.11490

Copyright © 2025 Eapen et al. This is an open-access publication distributed under the terms of the Creative Commons Attribution license.

advance HVCCC education. In 2021, two STARS students (Sruthi Eapen and Aylmer Tan) at Oregon Health & Science University (OHSU) embarked on integrating early HVCCC education into the existing curriculum with the goal of improving medical student knowledge and attitudes around HVCCC.

We grounded the curriculum in two theoretical frameworks: gamification to garner student buy-in, and spaced-education to promote knowledge retention. Gamification is defined as the selective incorporation of game elements into an educational or other non-game context.[5] Previous studies have found improved engagement, knowledge retention, and attitudes when gamification is incorporated into educational strategy.[6] In HVCCC education, the use of gamification was detailed in the *MedEdPORTAL* publication "The Bloody Board Game: A Game-Based Approach for Learning High-Value Care Principles in the Setting of Anemia Diagnosis."[7] This approach used a gameboard to teach high-value care and found improved medical knowledge and clinical reasoning utilizing HVCCC concepts for anemia workups. Spaced education involves repeated exposure to a concept spaced over time to improve retention.[8] In 2017, Matos et al. investigated using spaced clinical pearls to improve competency and retention of medical knowledge.[9]

Combining these two theoretical approaches, we integrated a gamified and spaced value-based care curriculum into existing platforms within the OHSU medical school curriculum. Previous *MedEdPORTAL* curricula have been developed for residents.[10] Our research builds on this by extending both the curriculum and the concept of gamification to medical students. We suspected that we could improve medical student HVCCC knowledge, attitudes, and competency using existing infrastructure and student-led content development to minimally impact the full medical school curriculum and limited faculty time.

Methods

OHSU's undergraduate medical education curriculum consists of an 18-month foundational phase with organ system-based blocks integrated into basic, clinical, and health systems sciences. This is followed by a second phase of core and elective clinical rotations. To integrate with the existing curricular framework, we approached the course director (Paul Gorman) for a clinical skills lab (CSL) taking place during the first 18-month preclinical phase of OHSU's curriculum. As a result, our primary audience consisted of first- and second-year medical students.

CSLs are weekly 90-minute faculty-led classes (with approximately ten students per group), where students learn clinical skills such as physical exams, systems science (including HVCCC), ethics, and a variety of other topics. CSLs are sometimes paired with clinical informatics pearls (CIPs), weekly asynchronous skill-based informatics assignments comprising a clinical scenario, brief instructional video, and related online tasks designed to supplement the content covered in that week's didactic. For example, while students learn about the cardiovascular system in class, the CSL would teach the physical exam skill, and the CIP would teach them how to order cardiac medications through the electronic medical record.

Curriculum Integration

Taking advantage of this existing structure within OHSU's curriculum, Sruthi Eapen and Aylmer Tan proposed to the CSL course director to rework three asynchronous CIPs and a single in-person CSL session to focus on HVCCC concepts. A single CSL that integrated HVCCC concepts via a case discussion already existed in the curriculum. We asked to rework this session using gamification to promote student buy-in. To incorporate HVCCC concepts into the CIPs, we asked to build clinical cases around the weekly CSL topic that correlated to the clinical content taught that week but have the activity focus on HVCCC. Knowing that the course director had limited bandwidth to build new curriculum, we proposed to create the content ourselves under the supervision of our STARS mentor (Andrea Smeraglio) and present the material for course director approval. Before our curriculum intervention, students had received one lecture on high-value care but had not participated in any other formal HVCCC education experiences.

CIP Implementation

We created three approximately 3-minute-long CIPs delivered asynchronously via online video. Each CIP explained an HVCCC concept, how to access the related materials or guidelines and gave an exemplar case. The exemplar case used the CSL topic of the week to maintain synchronicity with the weekly CSL. The three HVCCC CIPs were spaced at 1- to 5-week intervals prior to the gamified CSL.

Our first CIP instructed students on how to check standard-of-care guidelines for imaging indications using an imaging appropriateness website. We selected the American College of Radiology Appropriateness Criteria for students to determine if imaging was indicated.[11] We gave students a short clinical exemplar case involving a patient presenting with forgetfulness and sleep disturbance and instructed students to use the American College of Radiology Appropriateness Criteria to determine if imaging was indicated (Appendix A).

Copyright © 2025 Eapen et al. This is an open-access publication distributed under the terms of the Creative Commons Attribution license.

Our second CIP instructed students on how to look up costs of labs and medications using readily available resources such as OHSU's cost-of-service spreadsheet and GoodRx for medication costs.[12] The FAIR Health consumer tool was included for programs that do not have a cost spreadsheet.[13] We tasked students with looking up the costs of imaging and helping a hypothetical patient find cheaper options for their medications using GoodRx (Appendix B).

With the third CIP, we used a practical example to reinforce the thought processes involved in high-value care decision-making. We gave students a test patient in our electronic health record with a prepopulated visit and notes. We asked them to critique the assessment and plan from a HVCCC lens. Using Choosing Wisely guidelines as an example, we instructed students to utilize HVCCC resources to aid them with the assignment (Appendix C).[14] Students then determined whether screening was necessary and if it followed clinical guidelines.

Gamified CSL Implementation

Overview: We culminated our three CIPs in a single gamified CSL within the standard 90-minute time slot that allowed students to apply their HVCCC knowledge to a simulated case. We structured the CSL as a case competition with the goal of getting the correct diagnosis and developing a treatment plan using HVCCC frameworks at the lowest total cost. Group facilitators divided students into teams of two to three individuals. The game began with a hypothetical case of a young patient presenting with generalized swelling and hematuria. We gave students the basic information about the case and instructed them to order any other information needed (Appendix D). Ultimately, the team that correctly diagnosed post-streptococcal glomerulonephritis (PSGN) with the lowest cost won the game.

Costs: Each lab, imaging, and procedure had an independent designated monetary cost based on OHSU's cost-of-service spreadsheet. Each team tracked costs using their cost worksheet (Appendix E). If a team ordered a basic metabolic panel and complete blood count, that represented two tests, and the team was charged for each item ordered.

Time: Students ordered tests using a time-tiered structure where every test had an associated time delay to mimic real life where some tests come back fast (like a complete blood count), and other tests take longer (like an MRI or procedure). To simulate this, some basic tests were available immediately, but other more advanced tests were only available further into the game. Once a tier of tests became available, it remained open to order for the rest of the game. The total CSL took around 1.5 hours.

Facilitator role: The role of the facilitator in the room was to provide data based on ordering done by students. The facilitator visually presented and explained the time-tiered ordering structure using the accompanying PowerPoint slide (Appendix D). The facilitator had read the entire facilitator guide and did not provide information without an order (Appendix F). The facilitator was instructed to keep results secret from anyone other than the ordering team. This approach was chosen to help simulate real-world decision-making where answers are often unclear, encouraging students to engage in high-value care reasoning on their own without immediate feedback. The facilitator also guided a discussion at the end prompting students on how they used HVCCC frameworks during the game.

Cost worksheet: The cost worksheet (Appendix E) tracked what tests were ordered and when. Students ordered a test by writing it on their cost worksheet. At the end of the game, students tallied up the total cost to make the diagnosis on the cost worksheet.

How to win: The team with the correct diagnosis at the lowest total cost won. If no team produced the correct diagnosis, the team with the diagnosis on their differential won. If both teams had the diagnosis on their differential, the team that spent the least money won. Of note, there was the option to buy a second diagnosis slot that gave the team two guesses at the final diagnosis, although this was an intentionally expensive option.

Evaluation

To gauge changes in medical students' knowledge, attitudes, and comfort with HVCCC concepts we administered an IRB-approved (OHSU Study# 00024671, approved August 23, 2022) pre- and postcurricular survey (Appendix G). Knowledge questions were multiple choice with a predetermined correct answer. We designed these questions to assess the intended learning points from each CIP and gamified CSL session. Students were concurrently going through the nervous system didactic block, giving them a medical student-level understanding of a headache differential. We based attitude questions on a 4-point Likert-type scale adapted from the Maastricht HVCCC Attitude Questionnaire (MHAQ).[15] We queried student perceptions of the HVCCC curriculum with effectiveness questions. We told students to complete the survey before starting the first CIP and again after the gamified CSL session. The postsurvey also included questions rating the effectiveness of the curriculum and comfort with applying HVCCC concepts. We piloted our survey with four medical students consisting of third- and fourth-year students for feedback and improvements. After taking the survey, we instructed the students to answer a set of questions about the survey to elicit feedback. We compiled and reviewed the

Copyright © 2025 Eapen et al. This is an open-access publication distributed under the terms of the Creative Commons Attribution license.

responses from the pilot and made edits to the survey where appropriate.

Statistical Analysis

We only included students who had completed both the pre- and postsurveys in the analysis to allow for comparability. We scored knowledge questions by adding a point for each correct response selected and deducting a point for each wrong answer selected. We compared the total score for students' responses pre- and postcurriculum using a paired *t* test. We scored attitude questions on a 4-point Likert-type scale with scores 4 and 3 classified as *agree* and scores 1 and 2 classified as *disagree*. We scored effectiveness questions on a 5-point Likert scale with scores 5 and 4 classified as *agree* and 1-3 classified as *disagree*. We used descriptive statistics to compare the percentage of students who agreed with each statement before and after the intervention. We did not include data of students who did not respond or opted not to have their response included in our analysis.

Results

One hundred and twenty-three first-year medical students (out of 145 total first-year medical students) completed the presurvey and indicated they would like to participate in our educational innovation. Sixty-three students completed the postsurvey, and 54 students completed both. Overall, 42% of students agreed/strongly agreed that the curriculum was effective/strongly effective at promoting cost-effective care. Sixty-five percent of students agreed they would likely use these resources during their clinical rotations. We compared the percentages of students who agreed/strongly agreed with attitude statements about HVCCC before and after the curricular intervention in the Table. The largest change in student agreement (44% precurriculum to 59% postcurriculum) was for the statement *Physicians should change their clinical practices (e.g., ordering, prescribing) if the costs of care they provide is higher than colleagues who care for similar patients*. Precurriculum, 4% of students ($n = 2$) felt comfortable/very comfortable accessing resources to provide cost-effective care, which improved to 41% ($n = 22$) postcurriculum. We did not find a significant difference ($p = .143$) between pre and posttest responses regarding knowledge of HVCCC.

Discussion

Two first-year medical students developed an integrated HVCCC curriculum for their classmates that nested into the existing medical education structure. To promote student engagement and positive attitudes towards HVCCC, we used gamification in the simulated case and spaced learning to help familiarize and solidify HVCCC concepts. Specifically, three CIPs were spaced at 1- to 5-week intervals leading up to the gamified CSL, providing students multiple opportunities to familiarize themselves with HVCCC resources and ideas before engaging in application during the gamified CSL. Delivering these activities required approval and coordination with curriculum directors, teaching services, and additional supporting faculty. Planning was done far in advance, as the activities needed to be scheduled during the preclinical didactic curriculum, which had limited free openings. Once specific dates were reserved, the activities were modified to match synergistically with the subjects (i.e., organ systems) being taught. Ultimately, we demonstrated that it is feasible to introduce HVCCC concepts early in medical student education, utilizing existing curricular resources.

Our most notable educational outcome was increased student comfort in utilizing HVCCC resources from 4% to 41% with only 2 hours and 25 minutes of curricular contact time. Even more notable, we reason that our three 15-minute CIPs were responsible for this change in comfort as they specifically focused on HVCCC resources. The CIPs mirrored the existing CSL topic to maintain synchronicity with the curriculum. This method of inserting small interactive HVCCC activities into existing curricular material may be a successful way to build comfort with HVCCC

Table. Pre- and Postcurricular Intervention Attitude Question Survey Responses Assessing Student Level of Agreement ($n = 54$)

Statement[a]	Presurvey No. (%)	Postsurvey No. (%)
Physicians should try not to think about the cost to the health care system when making treatment decisions.	15 (28)	22 (41)
Physicians should be aware of the costs of the tests or treatments they recommend.	54 (100)	54 (100)
Physicians should talk to patients about the costs of care when discussing treatment options.	52 (96)	49 (91)
Physicians should change their clinical practices (e.g., ordering, prescribing) if the costs of care they provide is higher than colleagues who care for similar patients.	24 (44)	32 (59)
Physician clinical practices (e.g., ordering, prescribing) are key drivers of high health care costs.	19 (35)	24 (44)
Costs to society should be important in physician decisions to use or not to use an intervention.	27 (50)	32 (59)
It is unfair to ask physicians to be cost-conscious and still keep the welfare of their patients foremost in their minds.	24 (44)	21 (39)
Cost-effectiveness data should be used to determine what treatments are offered to patients.	31 (57)	33 (61)
Trying to contain costs is the responsibility of every physician.	38 (70)	44 (81)
Physicians should discuss cost efficiency of care with their patients.	45 (83)	46 (85)

[a]Rated on a 4-point Likert-type scale (1 = *strongly disagree*, 4 = *strongly agree*). A 3 or 4 indicated agreement.

Copyright © 2025 Eapen et al. This is an open-access publication distributed under the terms of the Creative Commons Attribution license.

skills and resources while minimizing curricular contact time and integrating into existing topics. Additionally, the majority of students (65%) indicated that they were likely to utilize these resources when they transition to clinical rotations. This suggests that students found enough value and utility in the resources to implement them in a clinical setting.

While we were able to show improved comfort in resource utilization, our short curriculum did not statistically change general attitudes or knowledge around HVCCC. This observation is likely due to a ceiling effect, with a large proportion of students already agreeing that physicians should be aware of costs and incorporate costs into discussions with patients. Rather, this curriculum was focused on developing skills to act on these beliefs by using available resources to incorporate cost information into clinical decisions. We integrated these specific skills in the gamified scenario where students could make decisions with cost information. Additionally, the brevity of the knowledge survey may have also limited the quality of our results.

Our curriculum modification innovation had several limitations. First, student participation was not universal. The baseline didactic curriculum is designed to be largely self-driven where students can achieve a passing grade without completing all assignments. Consequently, a portion of students opted not to complete the modified curricular activities. Similarly, in the current curriculum, students were able to drop/not complete two assignments per block, potentially contributing to the poor postsurvey response, which occurred at the end of the block. Second, effectively running the CSL depended on the small groups' individual facilitators, which caused inconsistency in the students' experiences. Anecdotally, some of the small group's games did not run as smoothly as others. In future iterations, making assignments mandatory and running the gamified session with the facilitators first might improve overall participation and student experience. Additionally, we do not have data on the impact of the previous HVCCC curriculum for comparison. Finally, while students indicated they were likely to utilize the learned HVCCC resources during clinical rotations, the scope of this educational innovation does not track if this behavior will occur. Future longitudinal studies will be necessary to determine the long-term implications of a curriculum that introduces and emphasizes HVCCC early in medical education.

Appendices

A. Clinical Informatics Pearl 1.docx

B. Clinical Informatics Pearl 2.docx

C. Clinical Informatics Pearl 3.docx

D. Gamified Clinical Skills Lab.pptx

E. Cost Worksheet.docx

F. Facilitator Guide.docx

G. Pre- and Postsurvey.docx

All appendices are peer reviewed as integral parts of the Original Publication.

Sruthi Eapen: First-Year Medical Student, Oregon Health & Science University School of Medicine; ORCID: https://orcid.org/0009-0003-0092-8829

Aylmer Tan: First-Year Medical Student, Oregon Health & Science University School of Medicine

Paul Gorman, MD: Professor Emeritus, Oregon Health & Science University School of Medicine

Gretchen Scholl, MC: EHR Informaticist, Oregon Health & Science University School of Medicine

Andrea Smeraglio, MD: Associate Professor, Internal Medicine, Oregon Health & Science University School of Medicine; Portland Veterans Administration Hospital

Disclosures
None to report.

Funding/Support
Dr. Andrea Smeraglio received funding as part of a grant from the Arnold Foundation to study the Students and Trainees Advocating for Resource Stewardship (STARS) program and how we teach high-value care education.

Ethical Approval
The Oregon Health & Science University Institutional Review Board deemed further review of this project not necessary.

Disclaimer
The views expressed in this article are those of the authors and not the official position of the Department of Veterans Affairs, the United States Government, or any affiliated institutions or funders.

References

1. Shrank WH, Rogstad TL, Parekh N. Waste in the US health care system: estimated costs and potential for savings. *JAMA*. 2019; 322(15):1501-1509. https://doi.org/10.1001/jama.2019.13978
2. Cassel CK, Guest JA. Choosing wisely: helping physicians and patients make smart decisions about their care. *JAMA*. 2012; 307(17):1801-1802. https://doi.org/10.1001/jama.2012.476

Copyright © 2025 Eapen et al. This is an open-access publication distributed under the terms of the Creative Commons Attribution license.

3. *Medical School Graduation Questionnaire: 2022 All Schools Summary Report*. Association of American Medical Colleges; 2022. Accessed January 2, 2025. https://www.aamc.org/media/62006/download

4. Erath A, Mitchell M, Salwi S, Liu Y, Sherry A. The sooner the better: high-value care education in medical school. *Acad Med*. 2019;94(11):1643-1645. https://doi.org/10.1097/ACM.0000000000002881

5. Brigham TJ. An introduction to gamification: adding game elements for engagement. *Med Ref Serv Q*. 2015;34(4):471-480. https://doi.org/10.1080/02763869.2015.1082385

6. van Gaalen AEJ, Brouwer J, Schönrock-Adema J, Bouwkamp-Timmer T, Jaarsma ADC, Georgiadis JR. Gamification of health professions education: a systematic review. *Adv Health Sci Educ Theory Pract*. 2021;26(2):683-711. https://doi.org/10.1007/s10459-020-10000-3

7. Pisano TJ, Santibanez V, Hernandez M, Patel D, Osorio G. The bloody board game: a game-based approach for learning high-value care principles in the setting of anemia diagnosis. *MedEdPORTAL*. 2020;16:11057. https://doi.org/10.15766/mep_2374-8265.11057

8. Kerfoot BP, Kearney MC, Connelly D, Ritchey ML. Interactive spaced education to assess and improve knowledge of clinical practice guidelines: a randomized controlled trial. *Ann Surg*. 2009;249(5):744-749. https://doi.org/10.1097/SLA.0b013e31819f6db8

9. Matos J, Petri CR, Mukamal KJ, Vanka A. Spaced education in medical residents: an electronic intervention to improve competency and retention of medical knowledge. *PLoS One*. 2017;12(7):e0181418. https://doi.org/10.1371/journal.pone.0181418

10. Woods S, Avery C, Bartlett K, et al. High-value care pediatric curriculum. *MedEdPORTAL*. 2015;11:10146. https://doi.org/10.15766/mep_2374-8265.10146

11. ACR Appropriateness Criteria. American College of Radiology. Accessed January 2, 2025. https://www.acr.org/Clinical-Resources/ACR-Appropriateness-Criteria

12. GoodRx. Accessed January 2, 2025. https://www.goodrx.com/

13. Healthcare Cost Estimator. FAIR Health Consumer. Accessed January 2, 2025. https://www.fairhealthconsumer.org/

14. Choosing Wisely Recommendations: Don't obtain comprehensive viral panel testing for patients who have suspected respiratory viral illnesses. American Academy of Family Physicians. Accessed January 2, 2025. https://www.aafp.org/pubs/afp/collections/choosing-wisely/533.html

15. Mordang SBR, Könings KD, Leep Hunderfund AN, Paulus ATG, Smeenk FWJM, Stassen LPS. A new instrument to measure high value, cost-conscious care attitudes among healthcare stakeholders: development of the MHAQ. *BMC Health Serv Res*. 2020;20:156. https://doi.org/10.1186/s12913-020-4979-z

Received: May 29, 2024
Accepted: November 14, 2024
Published: January 28, 2025

Copyright © 2025 Eapen et al. This is an open-access publication distributed under the terms of the Creative Commons Attribution license.
